# CLL cells cumulate genetic aberrations prior to the first therapy even in outwardly inactive disease phase

**DOI:** 10.1038/s41375-018-0255-1

**Published:** 2018-09-12

**Authors:** María Hernández-Sánchez, Jana Kotaskova, Ana E Rodríguez, Lenka Radova, David Tamborero, María Abáigar, Karla Plevova, Rocío Benito, Nikola Tom, Miguel Quijada-Álamo, Vasileos Bikos, Ana África Martín, Karol Pal, Alfonso García de Coca, Michael Doubek, Nuria López-Bigas, Jesús-María Hernández-Rivas, Sarka Pospisilova

**Affiliations:** 1 Hematology Department, Hospital Universitario Salamanca, Salamanca, Spain; 2 IBSAL, IBMCC-Cancer Research Center, University of Salamanca, Salamanca, Spain; 3 Center of Molecular Medicine, Central European Institute of Technology, Masaryk University, Brno, Czech Republic; 4 Center of Molecular Biology and Gene Therapy, Department of Internal Medicine – Hematology and Oncology, University Hospital Brno and Medical Faculty, Masaryk University, Brno, Czech Republic; 5 Research Programon Biomedical Informatics, IMIM Hospital del Mar Medical Research Institute and Universitat Pompeu Fabra, Barcelona, Spain; 6 Catalan Institution for Research and Advanced Studies (ICREA), Barcelona, Spain; 7 Hematology Department, Hospital Clínico Universitario of Valladolid, Valladolid, Spain

**Keywords:** Chronic lymphocytic leukaemia, Genetics research

Over the past few years, several large-scale studies using next-generation sequencing (NGS) of whole-genomes (WGS) and whole-exomes (WES) have defined the mutational landscape of chronic lymphocytic leukemia (CLL) [1–4]. NGS studies have also revealed the clonal heterogeneity in CLL and showed that clonal evolution contributes to the variability in clinical course among CLL patients [3]. Clonal evolution is considered a key condition in CLL progression and relapse after treatment. Most CLL cases are diagnosed during the inactive disease phase, genetic aberrations’ underlying progress in CLL activity leading to the need for therapy are poorly understood and should be explored. A large number of frequently mutated genes have been identified and several putative driver mutations likely to confer selective growth advantage to CLL tumor cells have been proposed [1–3]. In addition, clonal shifts between paired treatment-naïve and relapsed CLL samples have been reported due to pre-existing subclone expansion under therapeutic pressure, demonstrating that clonal evolution likely underlies CLL relapse [3, 5]. Nevertheless, there are still a limited amount of longitudinal WES studies analyzing consecutive CLL samples before treatment intervegntion [6]. The acquisition of driver mutations accompanied by selectively neutral passenger changes during disease prior to therapy influence is therefore poorly documented. Here, WES was performed on consecutive treatment-naïve samples of CLL patients from three groups with different disease course: Active disease (AD) group: patients with an active disease before the second analyzed time-point (TP2); Stable disease (SD) group: cases with a period of stable phase after diagnosis followed by progression within 3 years after; and Indolent disease (ID) group: those with a long-term stable indolent disease. Moreover, we applied a novel integrative bioinformatics tool called “Cancer Genome Interpreter” to identify driver mutations [7].

Thirty-five CLL patients were included in the WES study. In total, 70 tumor samples – (two tumor time-points (TP) for each patient) - as well as 26 matched germline samples, were sequenced. Three groups of patients were characterized based on the disease activity at the second TP: (i) AD group (*n* = 20); (ii) SD group (*n* = 6); (iii) ID group (*n* = 9). Sampling points and group definition details are shown in Fig. 1. The disease activity was assessed according to iwCLL guidelines [8]. Sample characteristics are summarized in Supplementary Table S1, sample processing and WES analysis are detailed in Supplemental Material. In order to distinguish driver from passenger mutations, the novel bioinformatics tool “Cancer Genome Interpreter” (CGI, https://www.cancergenomeinterpreter.org/home) was used; [7] defined driver mutations were consequently validated by deep-targeted sequencing (DTS), as described previously [9]. Moreover, FISH data from testing of four recurrent cytogenetic aberrations (del13q, trisomy 12, del11q and del17p) were available for all samples.

**Fig. 1:**
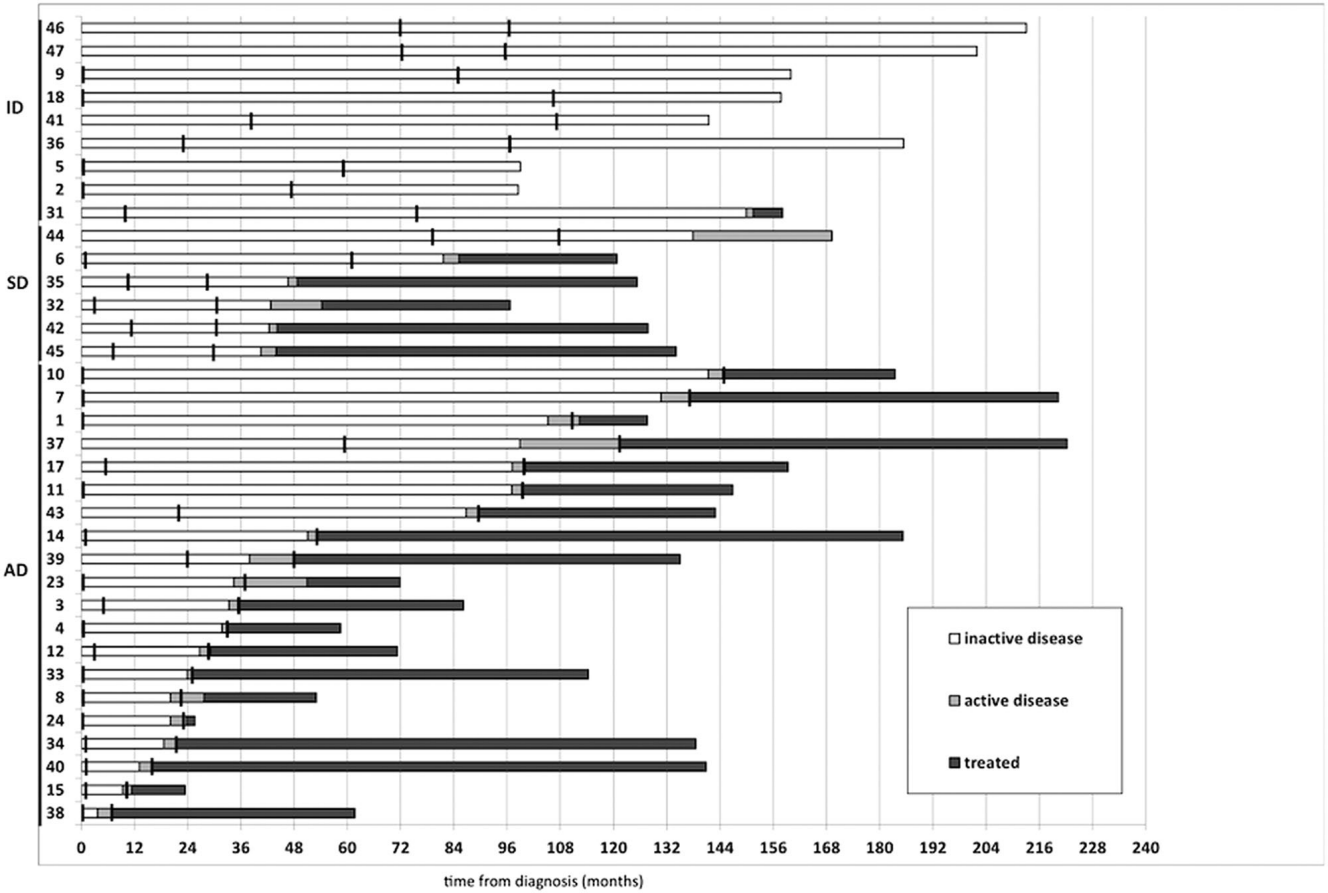
Sampling points and follow-ups of the tested cohort. Time-point 1 (TP1) for all tested samples was set to the inactive stage of the disease close to the diagnosis (median from diagnosis to TP1 = 2.1 months). Second time-points (TP2) were all collected prior to CLL-related therapy. Three groups of patients were then characterized based on disease activity in the TP2: (i) Active disease (AD) group - patients with TP2 in disease’s active phase (*n* = 20, median TTP = 33.9 months, median TTFT = 41.9 months); (ii) Stable disease (SD) group - patients with TP2 in disease’s inactive phase followed by active phase and therapy need (*n* = 6, median of time to progression (TTP) = 44.7 months, median of time to first treatment (TTFT) = 46.8 months); (iii) Indolent disease (ID) group - patients with TP2 taken in inactive phase, no disease activity or therapy need was reached during follow-up of 3 years (*n* = 9, median follow-up = 158.1 months). Only one ID case (P31) progressed after 150 months and required therapy intervention

WES analysis of samples from both TPs obtained from 26 CLL patients with available paired germline material showed presence of 25 somatic mutations. From WES analysis of 9 CLL patients with no available non-tumor control, 67 putatively somatic mutations were identified. Taken together, a total of 392 non-silent somatic or putatively somatic mutations (363 non-synonymous and 29 indels) were identified in 353 genes across the 35 CLL patients (Supplementary Table S2). Using CGI algorithm, 54 mutations were classified as “driver” and 338 mutations as “passenger” (Supplementary Table S2). The large majority of driver mutations (50/54, 92.6%) were further validated by deep-targeted sequencing (DTS) (Supplementary Table S3). Moreover, DTS of a 9-gene set recurrently mutated in CLL (*TP53, SF3B1, NOTCH1, NFKBIE, BIRC3, POT1, MYD88, XPO1, and EGR2)* revealed 7 mutations which were not detected by WES due to their low Variant Allele Frequency (VAF) (Supplementary Table S3). The 57 validated driver mutations were located in 35 different genes. The most frequently mutated genes were *SF3B1* (8/35, 22.9%), *NOTCH1* (4/35, 11.4%), *NFKBIE* (4/35, 11.4%), *TP53* (3/35, 8.6%), *BIRC3* (3/35, 8.6%), and *RPS15* (3/35, 8.6%) (Fig. 2). Among the other genes with a driver mutation, 11 had previously been reported as drivers in CLL patients [2, 3]. Additionally, CGI analysis also predicted driver mutations in *CDC73*, *DHX9*, *EGFR*, *ERCC6*, *FAT1*, *GATA3*, *G3BP1*, *HDAC2*, *IDH1*, and *PTCH1* genes that were unknown for CLL to date (Fig. 2). Among them, the tumor suppressor *FAT1* has been related to chemo-refractoriness in CLL [10]; *HDAC2* is known to be down-regulated in CLL [11]; and *DHX9*, *GATA3*, and *IDH1* have been described to be recurrently mutated in other hematological malignancies [12].

**Fig. 2:**
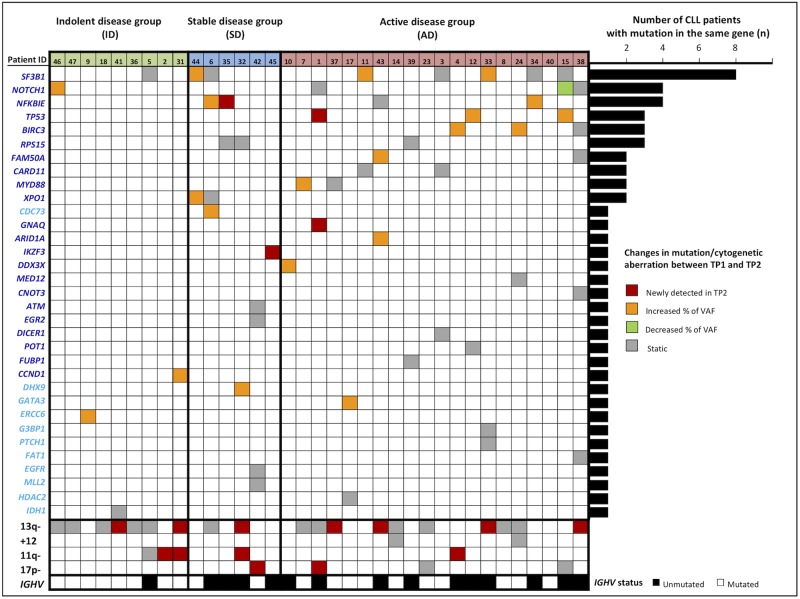
List of genes with driver mutations validated using deep targeted sequencing and their changes in variant allele frequency between time-points. Cut-off 5% of VAF was applied for validation of identified mutations. Allele frequency differences were tested across leukemia samples using a Fisher’s exact test. Mutations were considered to be changed if they were significantly different between samples (evolution *p*-value < 0.05) and their ratio of VAF between TP2 and TP1 was higher than 1.5 (for “increased” mutations) or lower than 0.375 (for “decreased” mutations). Genes with dark blue labels (on the left axis) were CLL drivers previously identified in Puente et al.^2^ and Landau et al.^3^ and, where those in light blue were unknown as CLL drivers to date

To identify somatic mutations which could be involved in clonal evolution, we analyzed the VAF dynamics between TP1 and TP2. Twenty-six out of 57 (46 %) driver mutations showed a significant change in allele frequency at the TP2: 4 were detected only at the TP2, 21 showed VAF increase at the TP2, and 1 mutation showed a decrease (Fig. 2). Additionally, FISH analysis of four recurrent cytogenetic aberrations at both TPs showed that 11/35 patients acquired one or more new cytogenetic alterations at TP2 (3/9 ID, 2/6SD, and 6/20 AD) (Fig. 2). The most often acquired aberration - deletion 13q, was detected in 7 cases (2/9 ID, 1/6SD, and 4/20 AD). Acquisition of deletion 11q was detected in 4 cases (2/9 ID, 1/6SD, and 1/20 AD). Two patients who acquired a 17p deletion were from the SD and AD group. Taking together the WES and FISH results, clonal evolution was observed in 5/9 ID patients, in 6/6SD patients and in 14/20 AD patients. Of note, 5/9 ID patients showed clonal evolution although they showed a long-term indolent disease (median follow-up = 158 months). Mutations in CLL drivers associated with aggressive clinical course such as *TP53*, *BIRC3, RPS15*, and *NFKBIE* [4, 13–15] were mostly detected within the AD/SD groups (Fig. 2). Nevertheless, there were well-known CLL driver mutations (*NOTCH1*, *SF3B1*) detected in two of eight ID patients, revealing the fact that the simple presence of such a mutation does not immediately lead to disease progression. Follow-up of these two patients with indolent disease already bearing a driver mutation at TP1 reached 99 (P5), and 213.1 (P46) months with no clinical evidence of disease activity to date as documented in Supplementary Table 1 (Fig. 1).

In summary, we performed a longitudinal study using whole-exome sequencing to characterize genetic alterations occurring during disease course before CLL-related therapy intervention in 35 CLL patients. We compared samples from indolent CLL to samples from a stable or active disease. To define potential driver mutations, we used novel integrative bioinformatics tool “Cancer Genome Interpreter”. We showed continual evolution with cytogenetic aberration and somatic mutation accumulation during the time prior to therapy intervention. Despite clonal evolution, including driver mutation presence in genes such as *NOTCH1* or *SF3B1*, observed in indolent CLL cases, there was no clinical evidence of disease activity during long-term follow-up after sampling. We conclude that the acquisition of aberrations is not limited to the active disease phase or relapses after therapy [3, 5, 6]. Moreover, mutational profiles of indolent or outwardly stable CLL cases show that the presence of CLL clones bearing driver mutations do not have to correspond directly with disease progression. Therefore, simple mutation acquisition does not necessarily lead to immediate disease progression; nevertheless, accumulating changes precede the manifestation of disease activity. In addition, clonal evolution can occur in the absence of adverse prognostic factors such as the presence of high-risk cytogenetic alterations or unmutated IGHV. In fact, the acquisition of mutations can happen in the absence of any FISH alterations (P35 or P45) as well as in IGHV-mutated CLLs (P46). Unfortunately, analysis of genomic changes does not fully explain the transformation to a more aggressive stage in all CLL patients (P40). It was reported that epigenetic changes could also fuel CLL evolution during disease progression [6]. Understanding CLL evolution from the time of diagnosis to therapy need may be essential to gain insight into the process of transformation from the initial inactive form to later more aggressive stages. Although white blood cells (WBC) count during disease course is more feasible than performing NGS studies, we have observed that the acquisition of genomic alterations does not have to simply correspond with an increase of WBC (P4 or 35). Then, genomic analysis should be made in larger longitudinal-based cohort studies in order to evaluate how to predict disease activation in CLL. On the other hand, to understand the genomic changes underlying CLL relapse, mutational analysis at the time of diagnosis may be irrelevant as additional aberrations may appear during time and clonal shifts are likely to happen. Such analysis should be done before therapy intervention to monitor tumoral clones that are responsible for CLL relapse.

## Electronic supplementary material

Supplementary Material

Supplementary Table S1

Supplementary Table S2

Supplementary Table S3
