# Supplementary material for: CLL cells cumulate genetic aberrations prior to the first therapy even in outwardly inactive disease phase
Source: Leukemia. 2018 Sep 12;33(2):518–58. doi: 10.1038/s41375-018-0255-1 (PMC6756121; doi:10.1038/s41375-018-0255-1)
Supplement: Supplementary file 1 — Supplementary Material [file 41375_2018_255_MOESM1_ESM.docx]

**Supplemental material**

**Patients**

Thirty-five CLL patients were included in the WES study (18 patients form *Hospital Universitario de Salamanca*, Spain; and 17 patients from University Hospital Brno, Czech Republic). The study was approved by the Ethical Committee of *Hospital Universitario de Salamanca*, as well as of University Hospital Brno. For all samples, written informed consents approved by the Ethics Committees of the respective institutions were available in accordance with the Declaration of Helsinki. Patients were diagnosed according to the revised World Health Organization criteria (1).

CLL cells were separated from peripheral blood using immunomagnetic separation Sample purity was verified by flow cytometry to be higher than 95%. From the Spanish cohort: CD19-positive B cells were purified by magnetically activated cell sorting (MACS) CD19 MicroBeads (Miltenyi Biotec, Bergisch Gladbach, Germany) resulting in a >95% purity, as analyzed by flow cytometry. In the Czech cohort, Each sample from the Brno cohort (patients 31 – 47) was processed in the same way. Non B-cells were depleted using bispecific antibodies during gradient centrifugation (RosetteSep™ Human B Cell Enrichment Cocktail, STEMCELL, Canada) as described previously (2). This way of separation enabled us to obtain high yield of unlabeled cells with purity over 95 % CLL cells (mostly over 98%).

**Whole-exome sequencing (WES)**

Genomic DNA from tumor samples as well as germline was sheared and used for the construction of sequencing library by TruSeq Exome Enrichment Kit (Illumina) and sequenced on an IlluminaHiSeq 2000.The bioinformatics pipeline for WES data analysis is further explained in the following article (3). Somatic variants were identified using a 2 × 3 χ2-test (chisq.test in R with permutation) across germline and both two tumor samples. Sites with asomatic p-value<0.05 and a germline frequency <0.10 were called somatic. For patients without corresponding non-tumor sample, a more severe filtering was performed removing variants with allele frequencies around 30-70% or 90-100% in both TPs, except for mutations previously reported in CLL. This may underestimate the number of somatic mutations in these samples; however, it will help to avoid the inclusion of rare germline mutations. All somatic mutations were covered by at least 10 reads and were further reviewed manually using the Integrative Genomics Viewer (4).

***In silico* analysis of driver and passenger mutations**

Driver mutations were identified by using the Cancer Genome Interpreter (CGI) pipeline (<https://www.cancergenomeinterpreter.org/home>). Briefly, mutations that are clinically or experimentally validated to drive tumor phenotypes –previously culled from public sources- are identified by the CGI, whereas the effect of the remaining variants of uncertain significance are *in silico* predicted by using the OncodriveMUT method. The main novelty of this tool with respect to other existing methods is the incorporation of features characterizing the genes (or regions within genes) where the mutations occur, derived from the analysis of large cohorts of tumors (6,792 samples across 28 cancer types) and samples from healthy donors (60,706 unrelated individuals). This knowledge (e.g. gene sites acting as hotspots of somatic mutations or protein domains depleted by functional variants in healthy samples) is combined with features that describe each variant (e.g. the consequence type of the mutation or its predicted functional impact) by using a set of heuristic rules to state the mutation as driver or passenger.

**Ultra-deep targeted sequencing and data analysis**

For validation of driver mutations, genomic DNA from tumor samples as well as germline DNA, where available, was amplified with high-fidelity Q5 Polymerase (New England Biolabs, Ipswich, MA, USA) using specific primers designed for affected genes regions identified by WES. The indexed library was prepared with Nextera XT DNA Sample Preparation Kit (Illumina, San Diego, CA, USA) and sequenced using MiSeq Reagent Kit v2 (300 cycles; Illumina, San Diego, CA, USA) on a MiSeq instrument according to manufacturer recommendations. In case of validated exchanges the coverage per tested base exceeded 1000 (≥1 000 in 92% of nucleotides); mean coverage reached 5063. In case of 9-gene set where whole exons were sequenced, the coverage per base exceeded 1000 (≥1 000 in 99.2% of exons); mean coverage reached 6658. For variant detection we used bioinformatics combining CLC Genomic Workbench version 7.5 (Qiagen, Hilden, Germany) and the deepSNV R-package (5-7). To analyze changes in VAF between time-points we used results from targeted NGS. To distinguish mutations with significant increase/decrease VAF in TP2 regarding TP1, we applied the Fisher exact test. Mutations with p<0.05 were taken as significantly increased/decreased between time-points.

**Supplementary references**

1. Swerdlow SH, Campo E, Pileri SA, Harris NL, Stein H, Siebert R, et al. The 2016 revision of the World Health Organization classification of lymphoid neoplasms. Blood. 2016;127(20):2375-90.

2. Kotaskova J, Tichy B, Trbusek M, Francova HS, Kabathova J, Malcikova J, et al. High expression of lymphocyte-activation gene 3 (LAG3) in chronic lymphocytic leukemia cells is associated with unmutated immunoglobulin variable heavy chain region (IGHV) gene and reduced treatment-free survival. J Mol Diagn. 2010;12(3):328-34.

3. DePristo MA, Banks E, Poplin R, Garimella KV, Maguire JR, Hartl C, et al. A framework for variation discovery and genotyping using next-generation DNA sequencing data. Nat Genet. 2011;43(5):491-8.

4. Robinson JT, Thorvaldsdottir H, Winckler W, Guttman M, Lander ES, Getz G, et al. Integrative genomics viewer. Nat Biotechnol. 2011;29(1):24-6.

5. Forbes SA, Beare D, Gunasekaran P, Leung K, Bindal N, Boutselakis H, et al. COSMIC: exploring the world's knowledge of somatic mutations in human cancer. Nucleic Acids Res. 2015;43(Database issue):D805-11.

6. Vandenbroucke I, Van Marck H, Verhasselt P, Thys K, Mostmans W, Dumont S, et al. Minor variant detection in amplicons using 454 massive parallel pyrosequencing: experiences and considerations for successful applications. Biotechniques. 2011;51(3):167-77.

7. Varley JM. Germline TP53 mutations and Li-Fraumeni syndrome. Hum Mutat. 2003;21(3):313-20.

**Supplemental table legends**

Supplementary Table S1: Overview of clinical and biological data of CLL patients included in the WES study.

Supplementary Table S2: Non-silent mutations detected by WES in CLL patients; selection based on *in silico* Cancer Genome Interpreter analysis.

Supplementary Table S3: Driver mutations detected by WES and validated using DTS in CLL patients; selection based on in silico Cancer Genome Interpreter analysis.
