# Supplementary material for: CLL cells cumulate genetic aberrations prior to the first therapy even in outwardly inactive disease phase
Source: Leukemia. 2018 Sep 12;33(2):518–58. doi: 10.1038/s41375-018-0255-1 (PMC6756121; doi:10.1038/s41375-018-0255-1)
Supplement: Supplementary file 2 — Supplementary Table S1 [file 41375_2018_255_MOESM2_ESM.pdf]

Table S1: Overview of clinical and biological data of CLL patients included in the WES study

							TP1								TP2						last follow-up							
**Group**	**Patient ID**	**Age at diagnosis (years)**	**Gender**	**Rai stage at diagnosis**	**Binet stage at diagnosis**	**IGHV**	**Time from diagnosis to TP1 (months)**	**FISH at TP1**	**WBC (*10^9/L) at TP1**	**% Lymphocytes at TP1**	**Lymphadenopathy/Hepatomegaly/Splenomegaly**	**Rai stage at TP1**	**Binet stage at TP1**	**Time from TP1 to TP2 (months)**	**FISH at TP2**	**WBC (*10^9/L) at TP2**	**% Lymphocytes at TP2**	**Lymphadenopathy/Hepatomegaly/Splenomegaly**	**Binet stage at TP2**	**TTP (months)**	**Progression**	**Lymphadenopathy/Hepatomegaly/Splenomegaly**	**Binet stage at last follow-up**	**TTFT (months)**	**Follow-up (months)**	**Alive /Dead**	**Genes with CLL driver mutations**	**Changes in mutation between TP1 and TP2**
indolent	2	37.6	M	0	A	mutated	0.6	normal	10.2	48.4	no	0	A	47.1	11q-	10.4	48.8	no	A	not reached	not reached	no	A	not reached	98.6	Alive	-	-
indolent	5	55.8	M	0	A	unmutated	0.0	13q-, 11q-	26.2	61	no	0	A	59.7	13q-, 11q-	40	65	no	A	not reached	not reached	no	A	not reached	99.0	Alive	*SF3B1*	static
indolent	9	63.5	M	0	A	mutated	1.1	normal	11.7	63	no	0	A	86.4	normal	10.6	57.4	no	A	not reached	not reached	no	A	not reached	160.0	Alive	-	-
indolent	18	54.3	M	0	A	mutated	0.0	13q-	25.2	79.2	no	0	A	104.1	13q-	21.5	74.3	no	A	not reached	not reached	no	A	not reached	157.8	Alive	-	-
indolent	36	43.2	M	0	A	mutated	29.2	13q-	16.5	61.3	no	0	A	72.0	13q-	39.4	75.7	no	A	not reached	not reached	no	A	not reached	185.4	Alive	-	-
indolent	41	51.4	M	0	A	mutated	45.2	normal	121	96.8	no	0	A	60.9	13q-	157	93.4	no	A	not reached	not reached	no	A	not reached	141.5	Dead (not CLL-related)	-	-
indolent	46	55.4	F	0	A	mutated	73.5	13q-	44.8	89.9	no	0	A	18.7	13q-	24.3	79.2	no	A	not reached	not reached	no	A	not reached	213.1	Alive	*NOTCH1*	increased
idolent	31	52.4	F	0	A	mutated	11.2	normal	13.2	62.5	no	0	A	63.2	11q-, 13q-	11.91	51	no	A	149.9	more than 6 years after TM2	NA	NA	151.5	158.1	Alive	-	-
indolent	47	47.8	F	0	A	mutated	78.2	13q-	18.8	78.3	no	0	A	15.6	13q-	17	65.3	no	A	not reached	not reached	no	A	not reached	201.9	Alive	-	-
median<br>(range)		52.4<br>(37.6 - 63.5)					11.2<br>(0 - 78.2)													not reached				not reached	158.1<br>(98.6 - 213.1)			
stable	6	52.5	F	0	A	unmutated	1.1	13q-	14.8	69.6	no	0	A	62.9	13q-	212	93.4	no	A	81.6	within 3 years after TP2	NA	NA	82.1	117.8	Alive	*SF3B1 , NFKBIE , XPO1*	static, increased, static
stable	32	48.7	F	0	A	unmutated	3.5	normal	13.92	45.7	no	0	A	37.4	11q-, 13q-	102	92	no	A	42.8	within 3 years after TP2	NA	NA	54.2	96.7	Alive	*RPS15*	static
stable	35	54.4	F	0	A	unmutated	13.6	normal	30.5	82.8	no	0	A	17.8	normal	35.7	89.2	no	A	46.7	within 3 years after TP2	NA	NA	46.8	123.3	Dead	*NFKBIE , RPS15*	acquired, static
stable	42	64.4	F	0	A	mutated	14.7	normal	24.3	95	no	0	A	23.5	17p-	54.8	97.4	no	A	42.4	within 3 years after TP2	NA	NA	44.2	127.8	Alive	*ATM , EGR2*	static, static
stable	44	55.0	F	0	A	mutated	90.7	normal	144	91.6	no	0	A	18.0	normal	157	95.5	no	A	137.9	within 3 years after TP2	NA	NA	not reached	169.2	Dead (not CLL-related)	*SF3B1 , XPO1*	increased, increased
stable	45	58.0	F	0	A	unmutated	9.0	normal	187	96.2	no	0	A	26.9	normal	429	96.2	no	A	40.6	within 3 years after TP2	NA	NA	41.0	131.1	Alive	*IKZF3*	acquired
median<br>(range)		54.7<br>(48.7-64.4)					11.3<br>(1.1 - 90.7)													44.7<br>(40.6 - 137.9)				46.8<br>(41.0 - 82.1)	125.5<br>(96.7 - 169.2)			
active	1	58.4	F	0	A	unmutated	0.0	13q-	18.9	75.3	no	0	A	106.1	17p-, 13q-	250.9	52.9	yes	B	105.3	before TP2	NA	NA	106.4	121.7	Dead (related to CLL)	*NOTCH1 , TP53 , GNAQ*	static, acquired, acquired
active	3	55.9	M	1	B	mutated	6.9	normal	32	80.4	yes	1	B	26.6	normal	64	90.3	yes	C	33.3	before TP2	NA	NA	35.7	86.2	Alive	*SF3B1 , CARD11 , DICER1*	static, static, static
active	4	52.9	M	2	A	unmutated	1.0	normal	12.8	45.5	yes	2	A	31.7	11q-	12.2	67	yes	B	31.7	before TP2	NA	NA	32.9	58.5	Alive	*BIRC3*	increased
active	7	37.8	M	1	A	mutated	0.0	13q-	18.1	65.9	yes	1	A	131.1	13q-	52.8	92.4	yes	B	130.7	before TP2	NA	NA	131.3	214.2	Alive	*MYD88*	increased
active	8	73.9	F	0	A	mutated	0.0	13q-	45.5	78.9	no	0	A	20.8	13q-	41	94.9	yes	C	20.1	before TP2	NA	NA	27.6	53.0	Dead (CLL-related)	-	-
active	10	56.1	M	0	A	unmutated	1.4	normal	14.9	61.1	no	0	A	140.5	normal	386	94.6	yes	A	141.3	before TP2	NA	NA	144.7	183.4	Alive	*DDX3X*	increased
active	11	56.8	F	0	A	mutated	0.1	normal	13.2	74.8	no	0	A	97.1	normal	203	97	yes	A	97.1	before TP2	NA	NA	97.6	144.8	Alive	*SF3B1 , CARD11*	increased, static
active	12	74.5	F	1	B	unmutated	4.1	normal	88	87.2	yes	1	B	22.8	normal	68	89.7	yes	B	26.7	before TP2	NA	NA	27.1	69.3	Dead (CLL-related)	*TP53 , POT1*	increased, static
active	14	54.1	F	0	A	mutated	0.6	13q-, 12+	11.9	76.8	no	0	A	51.3	13q-, 12+	25.6	87.1	yes	A	51.0	before TP2	NA	NA	53.3	185.3	Alive	-	-
active	15	69.6	F	1	A	unmutated	2.1	17p-	10.2	69.9	yes	1	A	7.4	17p-	10.4	76.8	yes	C	9.3	before TP2	NA	NA	9.4	21.5	Dead (CLL-related)	*SF3B1 , NOTCH1 , TP53*	static, decreased, increased
active	17	42.5	F	0	A	mutated	6.3	normal	12.8	75.8	no	0	A	91.4	normal	81.8	83.1	yes	A	97.2	before TP2	NA	NA	100.1	159.3	Alive	-	-
active	23	56.0	M	2	B	mutated	0.4	13q-, 17p-	20.2	76.7	yes	2	B	34.0	13q-, 17p-	47	88.7	yes	C	34.4	before TP2	NA	NA	51.0	71.8	Alive	-	-
active	24	74.8	F	0	A	mutated	0.0	13q-, 12+	96.5	89.4	no	0	A	20.6	13q-, 12+	305.9	91	yes	C	20.1	before TP2	NA	NA	20.8	22.6	Dead (CLL-related)	*BIRC3 , MED12*	increased, static
active	33	55.1	M	I	A	unmutated	0.5	normal	132	94,9	yes	I	A	24.1	13q-	217	96,4	yes	B	23.9	before TP2	NA	NA	25.1	114.3	Alive	*SF3B1*	increased
active	34	50.9	M	I	B	unmutated	2.9	normal	39.6	84.9	yes	I	B	17.5	normal	103	94.5	yes	B	18.6	before TP2	NA	NA	21.6	138.6	Alive	*SF3B1 , NFKBIE*	static, increased
active	37	49.6	M	I	A	mutated	72.6	normal	46.2	91.5	yes	I	A	47.5	13q-	339	96.8	yes	B	98.8	before TP2	NA	NA	121.3	222.2	Alive	MYD88	static
active	38	69.0	F	III	C	unmutated	0.2	normal	128	92.7	yes	III	C	6.7	13q-	144	94.4	yes	C	3.8	before TP2	NA	NA	6.8	61.6	Alive	*NOTCH1 , BIRC3 , FAM50A , CNO*	static, static, static, static
active	39	65.1	M	0	A	unmutated	39.6	normal	107	88.2	no	0	A	8.1	normal	259	91.3	yes	C	38.0	before TP2	NA	NA	48.1	135.0	Dead (CLL-related)	*RPS15 , FUBP1*	static, static
active	40	59.9	M	I	A	mutated	2.1	normal	24.8	89.6	yes	I	A	13.8	normal	36,5	90,3	yes	C	13.2	before TP2	NA	NA	16.0	140.8	Alive	-	-
active	43	52.1	M	0	A	unmutated	29.5	normal	10.2	40.6	no	0	A	58.3	13q-	140	92.7	yes	A	86.8	before TP2	NA	NA	89.6	143.0	Alive	*NFKBIE , FAM50A , ARID1A*	static, increased, increased
median<br>(range)		56.0<br>(37.6 - 63.5)					1.2<br>(0 - 72.6)													33.9<br>(3.8 - 141.3)				41,9<br>(6.8 - 144.7)	128.3<br>(21.5 - 222.2)			

TTP - time to progression
TTFT - time to first treatment
NA - not applicable
WBC - white blood cells
