# Supplementary material for: CLL cells cumulate genetic aberrations prior to the first therapy even in outwardly inactive disease phase
Source: Leukemia. 2018 Sep 12;33(2):518–58. doi: 10.1038/s41375-018-0255-1 (PMC6756121; doi:10.1038/s41375-018-0255-1)
Supplement: Supplementary file 3 — Supplementary Table S2 [file 41375_2018_255_MOESM3_ESM.pdf]

Table S2: Non-silent mutations detected by WES in CLL patients; selection based on *in silico* Cancer Genome Interpreter analysis

Patient ID	Group (Indolent/Stable/Active)	Chromosome	Position	Reference allele	Altered allele	Depth at TP1	Reference reads at TP1	Altered reads at TP1	Allele frequency TP1	Depth at TP2	Reference reads at TP2	Altered reads at TP2	Allele frequency TP2	Somatic TP1 p-value	Somatic TP2 p-value	Gene	Strand	Transcript	Genome change
2	Indolent	17	11556252	A	G	94	78	16	0.17	93	57	36	0.39	0.000272314	1.20E-09	*DNAH9*	+	ENST00000262442	chr17:g.11556252A>G
2	Indolent	4	20619160	T	G	95	86	9	0.10	100	54	46	0.46	0.019610816	2.98E-14	*SLIT2*	+	ENST00000504154	chr4:g.20619160T>G
2	Indolent	7	87445498	C	A	59	55	4	0.07	77	61	16	0.21	0.177914607	0.00016045	*RUNDC3B*	+	ENST00000338056	chr7:g.87445498C>A
2	Indolent	3	130159113	TGAGTTTAAG	T	104	78	26	0.29	148	106	42	0.28	6.45E-05	3.54E-05	*COL6A5*	+	ENST00000265379	chr3:g.130159115_130159123delAGTTTAAGG
2	Indolent	12	130919281	G	A	128	108	20	0.16	116	67	49	0.42	0.030222951	4.45E-11	*RIMBP2*	-	ENST00000261655	chr12:g.130919281G>A
5	Indolent	19	1231080	C	G	68	43	25	0.37	198	107	91	0.46	0.004858239	3.17E-06	*C19orf26*	-	ENST00000215376	chr19:g.1231080C>G
5	Indolent	10	12043768	C	G	45	20	25	0.57	150	79	71	0.47	7.23E-06	7.88E-06	*UPF2*	-	ENST00000356352	chr10:g.12043768C>G
5	Indolent	16	21139066	C	T	49	27	22	0.45	120	69	51	0.43	9.51E-06	1.33E-07	*DNAH3*	-	ENST00000261383	chr16:g.21139066C>T
5	Indolent	13	25671027	A	G	139	122	17	0.12	200	155	45	0.23	0.022109684	1.10821E-05	*PABPC3*	+	ENST00000281589	chr13:g.25671027A>G
5	Indolent	12	109017650	G	C	82	74	8	0.10	196	145	51	0.26	0.781782286	0.000477549	*SELPLG*	-	ENST00000228463	chr12:g.109017650G>C
5	Indolent	2	113074074	G	C	30	14	16	0.53	116	65	51	0.44	0.00024383	0.000301394	*ZC3H6*	+	ENST00000409871	chr2:g.113074074G>C
5	Indolent	1	156707534	C	T	168	129	39	0.06	58	54	4	0.23	1	0.006050565	*MRPL24*	-	ENST00000361531	chr1:g.156707534C>T
5	Indolent	2	198267371	G	C	18	10	8	0.44	87	37	50	0.57	0.307123745	0.012348647	*SF3B1*	-	ENST00000335508	chr2:g.198267371G>C
9	Indolent	19	917657	T	C	91	91	0	0.00	34	28	6	0.18	1	6.35E-05	*KISS1R*	+	ENST00000234371	chr19:g.917657T>C
9	Indolent	1	1509888	T	C	118	87	31	0.26	28	21	7	0.25	4.49E-15	2.39E-07	*SSU72*	-	ENST00000291386	chr1:g.1509888T>C
9	Indolent	19	8656852	C	A	133	105	28	0.21	56	30	26	0.46	1.22E-05	9.13E-11	*ADAMTS10*	-	ENST00000270328	chr19:g.8656852C>A
9	Indolent	12	8692492	G	A	143	116	27	0.19	70	45	25	0.36	8.94E-07	6.77E-11	*CLEC4E*	-	ENST00000299663	chr12:g.8692492G>A
9	Indolent	7	8790830	T	A	93	79	14	0.15	58	33	25	0.43	0.000920299	8.48E-10	*NXPH1*	+	ENST00000405863	chr7:g.8790830T>A
9	Indolent	15	32972047	A	T	104	81	23	0.22	64	42	22	0.34	3.06E-07	3.81E-10	*SCG5*	+	ENST00000300175	chr15:g.32972047A>T
9	Indolent	10	50736490	C	A	127	105	22	0.17	53	40	13	0.25	3.45E-06	7.28004E-07	*ERCC6*	-	ENST00000355832	chr10:g.50736490C>A
9	Indolent	5	89943443	G	T	118	63	55	0.47	73	33	40	0.55	2.16E-10	1.93E-11	*GPR98*	+	ENST00000405460	chr5:g.89943443G>T
9	Indolent	5	89943483	A	C	102	50	52	0.49	60	24	36	0.60	1.46E-11	5.39E-13	*GPR98*	+	ENST00000405460	chr5:g.89943483A>C
9	Indolent	12	100649934	C	A	61	50	11	0.18	49	34	15	0.31	0.002194924	1.89E-05	*DEPDC4*	-	ENST00000416321	chr12:g.100649934C>A
9	Indolent	5	158267056	C	T	95	80	15	0.16	52	35	17	0.33	1.60E-06	9.50E-11	*EBF1*	-	ENST00000313708	chr5:g.158267056C>T
18	Indolent	4	2664614	A	G	128	83	45	0.35	149	91	58	0.39	1.01E-09	8.53E-12	*FAM193A*	+	ENST00000382839	chr4:g.2664614A>G
18	Indolent	6	28269016	G	T	88	66	22	0.25	110	74	36	0.33	1.61E-05	1.11E-07	*PGBD1*	+	ENST00000405948	chr6:g.28269016G>T
18	Indolent	9	34179067	T	C	96	71	25	0.26	108	66	42	0.39	4.92E-13	7.45E-21	*UBAP1*	+	ENST00000545103	chr9:g.34179067T>C
18	Indolent	9	34179124	T	G	105	79	26	0.25	121	82	39	0.32	1.52E-08	2.47E-12	*UBAP1*	+	ENST00000545103	chr9:g.34179124T>G
18	Indolent	17	36483247	T	A	149	110	39	0.26	172	103	69	0.40	7.11E-05	5.09E-08	*GPR179*	-	ENST00000342292	chr17:g.36483247T>A
18	Indolent	12	59270256	C	G	83	60	23	0.28	104	62	42	0.40	5.96E-07	1.46E-11	*LRIG3*	-	ENST00000320743	chr12:g.59270256C>G
18	Indolent	10	68139039	G	C	84	69	15	0.18	110	79	31	0.28	2.50E-08	1.37E-14	*CTNNA3*	-	ENST00000433211	chr10:g.68139039G>C
18	Indolent	3	88040002	C	A	145	115	30	0.21	131	78	53	0.40	1.07E-07	2.16E-15	*HTR1F*	+	ENST00000319595	chr3:g.88040002C>A
18	Indolent	6	101247393	C	T	62	31	31	0.49	78	44	34	0.44	7.44E-07	2.36E-06	*ASCC3*	-	ENST00000369162	chr6:g.101247393C>T
18	Indolent	10	120916240	T	C	57	42	15	0.26	65	36	29	0.45	1.67E-06	7.43E-12	*SFXN4*	-	ENST00000355697	chr10:g.120916240T>C
18	Indolent	6	143093805	T	C	130	96	34	0.26	156	104	52	0.33	3.75E-13	5.06E-18	*HIVEP2*	-	ENST00000367603	chr6:g.143093805T>C
18	Indolent	5	176932018	G	A	195	143	52	0.27	223	127	96	0.45	1.17E-13	1.80E-27	*DOK3*	-	ENST00000357198	chr5:g.176932018G>A
31	Indolent	8	61743095	T	C	39	28	11	0.28	50	24	26	0.40	2.43E-05	1.14E-07	*CHD7*	+	ENST00000423902	chr8:g.61743095T>C
31	Indolent	11	69456187	G	A	181	129	52	0.29	143	105	38	0.27	1.44E-19	1.37E-16	*CCND1*	+	ENST00000227507	chr11:g.69456187G>A
31	Indolent	11	69456206	T	G	203	183	20	0.10	158	146	12	0.08	1.38E-06	1.09E-04	*CCND1*	+	ENST00000227507	chr11:g.69456206T>G
31	Indolent	4	73003836	A	C	12	9	3	0.25	19	13	6	0.28	8.39E-03	1.44E-03	*NPFFR2*	+	ENST00000308744	chr4:g.73003836A>C
31	Indolent	7	82584822	G	A	87	62	25	0.29	83	61	22	0.27	9.29E-09	9.58E-08	*PCLO*	-	ENST00000333891	chr7:g.82584822G>A
31	Indolent	11	113131005	T	G	51	38	13	0.26	47	34	13	0.28	5.45E-07	2.91E-07	*NCAM1*	+	ENST00000316851	chr11:g.113131005T>G
31	Indolent	3	134898707	C	T	59	45	14	0.24	56	44	12	0.21	1.10E-06	5.88E-06	*EPHB1*	+	ENST00000398015	chr3:g.134898707C>T
31	Indolent	5	148627364	G	A	115	85	30	0.26	103	75	28	0.27	7.97E-12	6.32E-12	*ABLIM3*	+	ENST00000506113	chr5:g.148627364G>A
31	Indolent	1	206822176	G	T	40	20	20	0.50	44	32	12	0.27	1.65E-09	3.38E-05	*DYRK3*	+	ENST00000367109	chr1:g.206822176G>T
36	Indolent	X	32305742	C	A	46	16	30	0.65	24	0	24	1.00	9.97E-14	1.21E-19	*DMD*	-	ENST00000357033	chrX:g.32305742C>A
36	Indolent	21	34948684	G	A	22	22	0	0.00	19	15	4	0.21	1	0.007427351	*SON*	+	ENST00000356577	chr21:g.34948684G>A
36	Indolent	7	57522860	T	C	58	34	24	0.41	41	22	19	0.46	9.60E-12	1.31E-11	*ZNF716*	+	ENST00000420713	chr7:g.57522860T>C
36	Indolent	10	79814381	C	A	167	119	48	0.28	138	76	62	0.45	3.84E-17	3.81E-27	*RPS24*	+	ENST00000440692	chr10:g.79814381C>A
36	Indolent	16	88495659	C	T	38	33	5	0.13	44	28	16	0.36	0.00067339	7.51E-11	*ZNF469*	+	ENST00000437464	chr16:g.88495659C>T
36	Indolent	2	109002778	C	T	26	16	10	0.38	31	19	12	0.39	0.000785585	0.000537561	*SULT1C4*	+	ENST00000272452	chr2:g.109002778C>T
36	Indolent	3	130712864	C	T	43	40	3	0.07	35	23	12	0.35	0.057181911	4.60E-07	*ATP2C1*	+	ENST00000393221	chr3:g.130712864C>T
36	Indolent	1	161024464	C	T	105	64	41	0.39	73	43	30	0.41	1.90E-18	5.66E-17	*ARHGAP30*	-	ENST00000368013	chr1:g.161024464C>T
36	Indolent	2	220355271	G	A	55	44	11	0.20	58	35	23	0.40	5.85E-07	2.74E-14	*SPEG*	+	ENST00000312358	chr2:g.220355271G>A
41	Indolent	9	13175812	T	G	84	37	47	0.55	80	42	38	0.48	1.84E-13	9.27E-11	*MPDZ*	-	ENST00000541718	chr9:g.13175812T>G
41	Indolent	4	26417188	GA	G	71	44	27	0.41	75	49	26	0.37	2.50E-10	1.81E-09	*RBPJ*	+	ENST00000342295	chr4:g.26417189delA
41	Indolent	19	44661280	C	T	144	75	69	0.48	227	108	119	0.52	1.32E-23	4.61E-29	*ZNF234*	+	ENST00000426739	chr19:g.44661280C>T
41	Indolent	19	51330248	G	A	163	85	78	0.48	164	89	75	0.46	3.55E-14	2.42E-13	*KLK15*	-	ENST00000598239	chr19:g.51330248G>A
41	Indolent	3	72957613	C	T	37	24	13	0.35	30	16	14	0.47	5.38E-07	6.30E-09	*GXYLT2*	+	ENST00000389617	chr3:g.72957613C>T
41	Indolent	16	75263636	C	T	150	69	81	0.54	176	86	90	0.51	3.01E-30	1.92E-29	*BCAR1*	-	ENST00000418647	chr16:g.75263636C>T
41	Indolent	8	77618149	C	G	321	237	84	0.26	319	224	95	0.30	3.58E-09	1.12E-10	*ZFHX4*	+	ENST00000521891	chr8:g.77618149C>G
41	Indolent	7	82544328	T	C	78	37	41	0.53	94	45	49	0.52	1.30E-10	7.62E-11	*PCLO*	-	ENST00000333891	chr7:g.82544328T>C
41	Indolent	12	99225848	C	T	47	28	19	0.38	37	20	17	0.47	3.78E-10	8.71E-12	*ANKS1B*	-	ENST00000547776	chr12:g.99225848C>T
41	Indolent	5	132272856	A	C	119	55	64	0.53	109	62	47	0.43	1.75E-18	3.69E-13	*AFF4*	-	ENST00000265343	chr5:g.132272856A>C
41	Indolent	X	142717015	CA	C	49	17	32	0.65	58	16	42	0.81	1.46E-10	1.82E-15	*SLITRK4*	-	ENST00000381779	chrX:g.142717020delA
41	Indolent	2	209104688	C	T	118	59	59	0.50	78	39	39	0.50	7.42E-15	2.2338E-13	*IDH1*	-	ENST00000415913	chr2:g.209104688C>T
46	Indolent	19	4511679	C	T	689	593	96	0.20	1151	985	166	0.20	NA	NA	*PLIN4*	-	ENST00000301286	chr19:g.4511679C>T
46	Indolent	1	111957562	C	G	17	13	4	0.24	31	30	1	0.03	NA	NA	*OVGP1*	-	ENST00000369732	chr1:g.111957562C>G
46	Indolent	2	167262652	A	G	140	108	32	0.22	194	107	87	0.45	NA	NA	*SCN7A*	-	ENST00000409855	chr2:g.167262652A>G
46	Indolent	3	195511334	T	C	3187	3050	137	0.20	6442	6141	301	0.16	NA	NA	*MUC4*	-	ENST00000463781	chr3:g.195511334T>C
47	Indolent	X	24228819	G	T	92	73	19	0.21	68	55	13	0.19	NA	NA	*ZFX*	+	ENST00000379177	chrX:g.24228819G>T
47	Indolent	13	25362145	G	T	48	35	13	0.27	38	15	23	0.61	NA	NA	*RNF17*	+	ENST00000255324	chr13:g.25362145G>T
47	Indolent	9	33386972	A	G	118	95	23	0.18	87	72	15	0.17	NA	NA	*AQP7*	-	ENST00000297988	chr9:g.33386972A>G
47	Indolent	4	85742665	T	G	53	48	5	0.09	36	28	8	0.22	NA	NA	*WDFY3*	-	ENST00000295888	chr4:g.85742665T>G
47	Indolent	10	96540295	C	T	49	39	10	0.20	39	30	9	0.23	NA	NA	*CYP2C19*	+	ENST00000371321	chr10:g.96540295C>T
47	Indolent	15	100252741	A	C	15	13	2	0.13	29	22	7	0.24	NA	NA	*MEF2A*	+	ENST00000354410	chr15:g.100252741A>C
47	Indolent	9	139272551	C	G	38	30	8	0.21	27	22	5	0.19	NA	NA	*SNAPC4*	-	ENST00000298532	chr9:g.139272551C>G
47	Indolent	9	139272560	A	G	38	31	7	0.18	26	23	3	0.12	NA	NA	*SNAPC4*	-	ENST00000298532	chr9:g.139272560A>G
47	Indolent	1	144866673	G	A	359	276	83	0.21	300	242	58	0.17	NA	NA	*PDE4DIP*	-	ENST00000369356	chr1:g.144866673G>A
47	Indolent	3	195511412	T	A	1646	1211	435	0.20	1790	1322	468	0.21	NA	NA	*MUC4*	-	ENST00000463781	chr3:g.195511412T>A
6	Stable	20	3296432	C	T	20	19	1	0.05	38	22	16	0.42	1	0.001603041	*C20orf194*	-	ENST00000252032	chr20:g.3296432C>T
6	Stable	18	7037669	G	A	201	191	10	0.06	288	180	108	0.40	1	2.71E-12	*LAMA1*	-	ENST00000389658	chr18:g.7037669G>A
6	Stable	16	23200778	G	A	38	38	0	0.00	59	35	24	0.41	1	0.000306952	*SCNN1G*	+	ENST00000300061	chr16:g.23200778G>A
6	Stable	1	23847478	G	A	141	118	23	0.16	223	112	111	0.49	0.438349969	9.03E-15	*E2F2*	-	ENST00000361729	chr1:g.23847478G>A
6	Stable	18	31318822	A	G	46	26	20	0.43	112	67	45	0.40	9.69E-05	1.68E-05	*ASXL3*	+	ENST00000269197	chr18:g.31318822A>G
6	Stable	20	34582984	TTGGA	T	144	102	42	0.30	223	142	81	0.41	1.54E-13	6.22E-21	*CNBD2*	+	ENST00000349339	chr20:g.34582985_34582988delTGGA

6	Stable	20	34582990	CT	C	144	102	42	0.27	223	142	81	0.40	1.49E-13	4.04E-22	*CNBD2*	+	ENST00000349339	chr20:g.34582992delT
6	Stable	20	34582993	GGGGCCTCCCCTTCCTAC	G	144	102	42	0.26	223	142	81	0.39	1.82E-13	9.96E-23	*CNBD2*	+	ENST00000349339	chr20:g.34582994_34583010delGGGCCTCCCCTTCCTAC
6	Stable	1	52933928	G	A	50	48	2	0.05	63	38	25	0.40	1	0.000429068	*ZCCHC11*	-	ENST00000257177	chr1:g.52933928G>A
6	Stable	1	54059812	A	G	40	38	2	0.05	81	51	30	0.37	1	0.014112923	*GLIS1*	-	ENST00000312233	chr1:g.54059812A>G
6	Stable	X	129354379	G	A	40	40	0	0.00	47	32	15	0.32	1	0.008782393	*ZNF280C*	-	ENST00000370978	chrX:g.129354379G>A
6	Stable	2	178346871	A	G	40	32	8	0.20	54	28	26	0.48	0.559575054	0.000231693	*AGPS*	+	ENST00000264167	chr2:g.178346871A>G
6	Stable	1	193111038	G	C	37	37	0	0.00	71	43	28	0.39	1	0.010074323	*CDC73*	+	ENST00000367435	chr1:g.193111038G>C
6	Stable	1	193121534	C	G	64	53	11	0.17	119	63	56	0.47	0.006082522	2.47293E-09	*CDC73*	+	ENST00000367435	chr1:g.193121534C>G
6	Stable	2	198267360	T	G	32	30	2	0.06	74	48	26	0.35	1	0.006617547	*SF3B1*	-	ENST00000335508	chr2:g.198267360T>G
6	Stable	2	198482574	T	G	52	23	29	0.44	65	30	35	0.54	4.04E-05	4.71E-08	*RFTN2*	-	ENST00000295049	chr2:g.198482574T>G
32	Stable	4	1388344	TCA	T	368	321	47	0.21	430	361	69	0.19	0.003957918	0.006644934	*CRIPAK*	+	ENST00000324803	chr4:g.1388348_1388349delAC
32	Stable	19	1440432	C	T	172	153	19	0.11	206	189	17	0.08	8.42E-08	2.91E-06	*RPS15*	+	ENST00000586686	g.chr19:1440432C>T
32	Stable	9	21971137	T	G	29	19	10	0.32	12	4	8	0.67	NA	NA	*CDKN2A*	-	ENST00000304494	chr9:g.21971137T>G
32	Stable	19	38876508	A	G	32	26	6	0.16	24	18	6	0.25	NA	NA	*GGN*	-	ENST00000334928	chr19:g.38876508A>G
32	Stable	6	44280885	A	C	26	14	12	0.48	43	22	21	0.49	0.026003973	0.012241433	*AARS2*	-	ENST00000244571	chr6:g.44280885A>C
32	Stable	1	182835593	T	G	87	86	1	0.01	80	64	16	0.20	1	0.005682012	*DHX9*	+	ENST00000367549	chr1:g.182835593T>G
35	Stable	19	1440414	C	T	214	121	93	0.43	212	122	90	0.44	5.31E-30	2.02647E-30	*RPS15*	+	ENST00000586686	chr19:g.1440414C>T
35	Stable	18	18564436	C	T	97	68	29	0.30	82	52	30	0.37	2.01E-07	5.90E-09	*ROCK1*	-	ENST00000399799	chr18:g.18564436C>T
35	Stable	19	22497246	A	G	128	105	23	0.18	131	105	26	0.20	0.049032741	0.018157876	*ZNF729*	+	ENST00000601693	chr19:g.22497246A>G
35	Stable	19	22497252	A	G	135	106	29	0.21	137	107	30	0.22	0.010609393	0.01059741	*ZNF729*	+	ENST00000601693	chr19:g.22497252A>G
35	Stable	1	35351679	C	T	69	41	28	0.40	80	39	41	0.51	0.001878461	5.08E-05	*DLGAP3*	-	ENST00000373347	chr1:g.35351679C>T
35	Stable	17	36622709	C	T	121	54	67	0.55	128	62	66	0.51	0.007031328	0.01791783	*ARHGAP23*	+	ENST00000431231	chr17:g.36622709C>T
35	Stable	5	41049478	G	A	111	76	35	0.32	138	72	66	0.48	5.23E-07	1.57E-11	*HEATR7B2*	-	ENST00000399564	chr5:g.41049478G>A
35	Stable	20	43132615	A	T	52	31	21	0.39	47	24	23	0.49	0.000158691	1.56E-05	*SERINC3*	-	ENST00000342374	chr20:g.43132615A>T
35	Stable	18	45555666	T	TGAGGCCGGC	73	58	15	0.21	116	91	25	0.22	0.006695372	0.008403746	*ZBTB7C*	-	ENST00000588982	chr18:g.45555670_45555678dupGCCGGCGAG
35	Stable	3	53781379	G	A	31	25	6	0.20	34	20	14	0.41	0.026591364	0.000118302	*CACNA1D*	+	ENST00000288139	chr3:g.53781379G>A
35	Stable	15	75651433	C	T	170	101	69	0.41	198	115	83	0.42	3.66E-26	3.61E-28	*MAN2C1*	-	ENST00000267978	chr15:g.75651433C>T
35	Stable	1	86167927	T	C	86	73	13	0.15	81	66	15	0.19	0.009554979	0.00269156	*ZNHIT6*	-	ENST00000370574	chr1:g.86167927T>C
35	Stable	3	164907477	A	T	91	60	31	0.34	117	58	59	0.51	1.43E-05	5.06E-09	*SLITRK3*	-	ENST00000475390	chr3:g.164907477A>T
42	Stable	19	1912206	G	C	106	64	42	0.40	68	38	30	0.44	0.029927665	0.013582232	*ADAT3*	+	ENST00000329478	chr19:g.1912206G>C
42	Stable	12	14993415	C	T	61	33	28	0.44	60	29	31	0.52	7.30E-05	9.73E-06	*ART4*	-	ENST00000228936	chr12:g.14993415C>T
42	Stable	X	17768111	C	A	86	44	42	0.49	69	40	29	0.42	1.71E-07	5.00E-06	*SCML1*	+	ENST00000380041	chrX:g.17768111C>A
42	Stable	22	23523221	T	A	38	21	17	0.42	42	21	21	0.50	0.000353247	1.47E-05	*BCR*	+	ENST00000305877	chr22:g.23523221T>A
42	Stable	2	26951420	G	A	160	109	51	0.31	129	73	56	0.43	2.72E-06	2.36E-09	*KCNK3*	+	ENST00000302909	chr2:g.26951420G>A
42	Stable	22	31529168	C	T	172	93	79	0.46	153	74	79	0.52	3.33E-22	7.98E-25	*INPP5J*	+	ENST00000404390	chr22:g.31529168C>T
42	Stable	15	34102839	C	T	65	30	35	0.56	49	24	25	0.51	6.79E-12	9.30E-10	*RYR3*	+	ENST00000389232	chr15:g.34102839C>T
42	Stable	21	42629239	C	T	29	15	14	0.46	36	17	19	0.53	0.006749326	0.001385782	*BACE2*	+	ENST00000330333	chr21:g.42629239C>T
42	Stable	17	45663749	G	A	31	27	4	0.14	37	27	10	0.27	0.353344768	0.018432745	*NPEPPS*	+	ENST00000322157	chr17:g.45663749G>A
42	Stable	12	49444932	C	CG	180	125	55	0.50	183	132	51	0.46	4.45E-07	1.63693E-06	*MLL2*	-	ENST00000301067	chr12:g.49444938dupG
42	Stable	7	55268023	G	A	19	8	11	0.58	22	12	10	0.45	0.001559605	0.012901562	*EGFR*	+	ENST00000275493	chr7:g.55268023G>A
42	Stable	12	58002424	C	T	82	41	41	0.50	73	35	38	0.52	1.93E-17	1.43E-17	*DTX3*	+	ENST00000548198	chr12:g.58002424C>T
42	Stable	11	58978209	C	A	51	29	22	0.43	50	24	26	0.52	6.23E-05	3.00E-06	*MPEG1*	-	ENST00000361050	chr11:g.58978209C>A
42	Stable	10	64573332	C	T	259	127	132	0.50	206	111	95	0.47	4.58E-12	4.08939E-11	*EGR2*	-	ENST00000242480	chr10:g.64573332C>T
42	Stable	11	64684532	C	T	64	37	27	0.42	111	48	63	0.57	8.93E-14	7.47E-25	*ATG2A*	-	ENST00000377264	chr11:g.64684532C>T
42	Stable	9	95021221	C	G	129	64	65	0.51	148	56	92	0.62	2.98E-16	3.36E-22	*IARS*	-	ENST00000375643	chr9:g.95021221C>G
42	Stable	8	101730010	T	C	67	54	13	0.13	69	49	20	0.25	0.100321999	0.002437148	*PABPC1*	-	ENST00000318607	chr8:g.101730010T>C
42	Stable	11	108043841	G	A	30	16	14	0.47	43	25	18	0.42	9.08E-05	0.000226621	*NPAT*	-	ENST00000278612	chr11:g.108043841G>A
42	Stable	11	108213973	G	A	22	13	9	0.43	35	19	16	0.46	0.001952253	0.000812256	*ATM*	+	ENST00000278616	chr11:g.108213973G>A
42	Stable	11	117222609	C	T	32	18	14	0.44	60	34	26	0.43	8.35E-05	1.73E-05	*CEP164*	+	ENST00000278935	chr11:g.117222609C>T
42	Stable	4	134073481	T	C	88	52	36	0.41	84	45	39	0.46	3.33E-05	4.88E-06	*PCDH10*	+	ENST00000264360	chr4:g.134073481T>C
42	Stable	4	154504360	T	C	51	27	24	0.49	72	46	26	0.36	6.15E-07	3.08E-05	*KIAA0922*	+	ENST00000409959	chr4:g.154504360T>C
42	Stable	1	177245476	C	A	68	26	42	0.60	63	31	32	0.51	6.25E-15	5.04E-12	*FAM5B*	+	ENST00000361539	chr1:g.177245476C>A
44	Stable	2	9661379	A	T	53	26	27	0.50	79	46	33	0.39	1.65E-13	8.80E-12	*ADAM17*	-	ENST00000310823	chr2:g.9661379A>T
44	Stable	8	17218646	C	T	24	12	12	0.50	26	13	13	0.50	0.000104245	0.000129703	*MTMR7*	-	ENST00000180173	chr8:g.17218646C>T
44	Stable	9	18776945	G	T	116	59	57	0.50	158	79	79	0.50	1.53E-10	3.06E-11	*ADAMTSL1*	+	ENST00000380548	chr9:g.18776945G>T
44	Stable	22	28395181	G	A	234	141	93	0.41	251	132	119	0.51	1.09E-29	1.79E-38	*TTC28*	-	ENST00000397906	chr22:g.28395181G>A
44	Stable	18	31432987	A	C	31	16	15	0.48	29	14	15	0.48	6.46E-06	8.19E-06	*NOL4*	-	ENST00000261592	chr18:g.31432987A>C
44	Stable	20	34611605	C	T	52	23	29	0.55	71	46	25	0.34	3.28E-05	0.017586963	*CNBD2*	+	ENST00000349339	chr20:g.34611605C>T
44	Stable	8	41552199	C	T	301	160	141	0.46	339	159	180	0.55	7.83E-31	2.98E-39	*ANK1*	-	ENST00000265709	chr8:g.41552199C>T
44	Stable	15	43632485	A	G	59	48	11	0.19	63	49	14	0.22	2.56E-05	2.09E-06	*ADAL*	+	ENST00000428046	chr15:g.43632485A>G
44	Stable	19	58774095	G	A	78	41	37	0.47	128	59	69	0.54	0.000379887	1.04E-05	*ZNF544*	+	ENST00000269829	chr19:g.58774095G>A
44	Stable	17	61559937	G	A	285	151	134	0.49	397	191	206	0.50	1.44E-25	6.00E-26	*ACE*	+	ENST00000290866	chr17:g.61559937G>A
44	Stable	2	61705998	C	T	27	20	7	0.26	23	16	7	0.30	0.003829079	0.001832476	*XPO1*	-	ENST00000401558	chr2:g.61705998C>T
44	Stable	4	77818320	G	A	67	59	8	0.12	87	69	18	0.21	0.009441067	0.000155334	*SOWAHB*	-	ENST00000334306	chr4:g.77818320G>A
44	Stable	13	88329213	G	A	29	16	13	0.45	51	30	21	0.41	0.016060653	0.011294351	*SLITRK5*	+	ENST00000325089	chr13:g.88329213G>A
44	Stable	8	90990521	T	C	20	10	10	0.50	35	22	13	0.37	0.004339394	0.020584094	*NBN*	-	ENST00000265433	chr8:g.90990521T>C
44	Stable	X	105855539	T	C	149	86	63	0.43	228	111	117	0.52	2.16E-11	1.51E-15	*CXorf57*	+	ENST00000372548	chrX:g.105855539T>C
44	Stable	8	110393640	G	T	40	21	19	0.48	51	27	24	0.47	0.00305351	0.002240718	*PKHD1L1*	+	ENST00000378402	chr8:g.110393640G>T
44	Stable	7	111584866	C	T	34	26	8	0.24	56	30	26	0.46	0.006289172	2.39E-06	*DOCK4*	-	ENST00000437633	chr7:g.111584866C>T
44	Stable	X	153044463	C	T	368	185	183	0.51	443	241	202	0.43	6.19E-37	3.01E-29	*PLXNB3*	+	ENST00000538966	chrX:g.153044463C>T
44	Stable	1	169100623	A	T	50	24	26	0.52	49	22	27	0.55	6.91E-09	2.04E-09	*ATP1B1*	+	ENST00000367816	chr1:g.169100623A>T
44	Stable	3	183273257	A	T	72	35	37	0.51	77	37	40	0.52	2.96E-13	1.90048E-13	*KLHL6*	-	ENST00000341319	chr3:g.183273257A>T
44	Stable	1	196295914	C	G	46	26	20	0.43	52	29	23	0.44	0.021545616	0.022085396	*KCNT2*	-	ENST00000294725	chr1:g.196295914C>G
45	Stable	19	1567568	C	A	48	28	20	0.43	36	17	19	0.53	7.95E-14	2.01E-15	*MEX3D*	-	ENST00000388824	chr19:g.1567568C>A
45	Stable	2	3627700	CCGTACT	C	27	21	6	0.16	33	19	14	0.50	0.114195545	7.75E-05	*RPS7*	+	ENST00000304921	chr2:g.3627702_3627707delGTACTC
45	Stable	17	9586182	G	A	120	64	56	0.47	121	59	62	0.52	9.11E-25	7.94E-28	*USP43*	+	ENST00000285199	chr17:g.9586182G>A
45	Stable	9	18753462	C	T	83	40	43	0.52	87	48	39	0.45	5.78E-19	2.40E-16	*ADAMTSL1*	+	ENST00000380548	chr9:g.18753462C>T
45	Stable	9	35396857	C	G	78	44	34	0.43	88	42	46	0.52	4.71E-21	3.67E-28	*UNC13B*	+	ENST00000378495	chr9:g.35396857C>G
45	Stable	19	36002395	C	T	28	22	6	0.21	26	20	6	0.23	0.041003223	0.036721261	*DMKN*	-	ENST00000339686	chr19:g.36002395C>T
45	Stable	17	37947776	A	C	51	48	3	0.06	50	39	11	0.22	0.075346942	4.30524E-05	*IKZF3*	-	ENST00000346872	chr17:g.37947776A>C
45	Stable	12	50513965	G	A	72	56	16	0.22	94	76	18	0.19	0.000434525	0.000802816	*COX14*	+	ENST00000550487	chr12:g.50513965G>A
45	Stable	11	56431607	A	T	120	109	11	0.09	118	92	26	0.22	0.01681061	4.24E-06	*OR5AR1*	+	ENST00000302969	chr11:g.56431607A>T
45	Stable	8	88365930	G	C	54	39	15	0.28	50	25	25	0.50	8.80E-05	8.37E-09	*CNBD1*	+	ENST00000518476	chr8:g.88365930G>C
45	Stable	10	88683360	G	A	168	85	83	0.49	191	100	91	0.47	5.50E-24	1.04E-23	*BMPR1A*	+	ENST00000224764	chr10:g.88683360G>A
45	Stable	6	99771341	G	A	19	12	7	0.37	23	10	13	0.57	0.002241266	2.25E-05	*FAXC*	-	ENST00000389677	chr6:g.99771341G>A
45	Stable	5	127474301	A	T	35	24	11	0.31	42	25	17	0.40	3.72E-06	1.55E-08	*SLC12A2*	+	ENST00000262461	chr5:g.127474301A>T
45	Stable	8	144511979	G	C	16	16	0	0.00	16	12	4	0.25	1	0.03912608	*MAFA*	-	ENST00000333480	chr8:g.144511979G>C
45	Stable	2	165984284	C	T	66	32	34	0.52	83	47	36	0.43	2.02E-05	0.000366403	*SCN3A*	-	ENST00000283254	chr2:g.165984284C>T
45	Stable	2	220309864	C	T	51	27	24	0.47	52	23	29	0.56	6.85E-09	8.04E-11	*SPEG*	+	ENST00000312358	chr2:g.220309864C>T
1	Active	18	580508	C	T	67	54	13	0.19	19	12	7	0.33	NA	NA	*CETN1*	+	ENST00000327228	chr18:g.580508C>T
1	Active	17	7577096	T	C	71	71	0	0.00	23	10	13	0.52	NA	NA	*TP53*	-	ENST00000269305	chr17:g.7577096T>C
1	Active	1	17272778	G	A	399	308	91	0.24	158	138	20	0.12	NA	NA	*CROCC*	+	ENST00000375541	chr1:g.17272778G>A
1	Active	14	21424360	C	T	72	71	1	0.01	32	20	12	0.39	NA	NA	*RNASE2*	+	ENST00000304625	chr14:g.21424360C>T
1	Active	8	21925137	G	A	200	153	47	0.23	63	28	35	0.55	NA	NA	*EPB49*	+	ENST00000523266	chr8:g.21925137G>A
1	Active	22	29446528	G	T	98	74	24	0.24	25	13	12	0.46	NA	NA	*ZNRF3*	+	ENST00000544604	chr22:g.29446528G>T
1	Active	X	30737625	G	A	116	91	25	0.22	42	23	19	0.54	NA	NA	*GK*	+	ENST00000378943	chrX:g.30737625G>A

1	Active	3	49167138	T	C	126	96	30	0.24	64	43	21	0.31	NA	NA	*LAMB2*	-	ENST00000418109	chr3:g.49167138T>C
1	Active	15	54307135	G	C	50	50	0	0.00	13	7	6	0.45	NA	NA	*UNC13C*	+	ENST00000260323	chr15:g.54307135G>C
1	Active	9	80412493	C	T	65	65	0	0.00	31	14	17	0.55	NA	NA	*GNAQ*	-	ENST00000286548	chr9:g.80412493C>T
1	Active	X	91133813	C	A	156	136	20	0.13	120	63	57	0.47	NA	NA	*PCDH11X*	+	ENST00000373094	chrX:g.91133813C>A
1	Active	10	115411639	G	A	126	126	0	0.00	51	29	22	0.42	NA	NA	*NRAP*	-	ENST00000359988	chr10:g.115411639G>A
1	Active	6	116837115	C	T	161	141	20	0.12	35	17	18	0.55	NA	NA	*FAM26E*	+	ENST00000368599	chr6:g.116837115C>T
1	Active	9	139390648	CAG	C	91	81	10	0.07	49	27	22	0.48	NA	NA	*NOTCH1*	-	ENST00000277541	chr9:g.139390649_139390650delAG
1	Active	4	187534356	C	T	57	47	10	0.18	26	15	11	0.46	NA	NA	*FAT1*	-	ENST00000441802	chr4:g.187534356C>T
3	Active	7	2978389	T	G	124	69	55	0.44	104	44	60	0.58	1.38E-27	3.30386E-35	*CARD11*	-	ENST00000396946	chr7:g.2978389T>G
3	Active	19	6733606	C	G	51	44	7	0.14	42	26	16	0.38	0.000217492	7.80E-11	*GPR108*	-	ENST00000264080	chr19:g.6733606C>G
3	Active	14	95574253	C	T	81	36	45	0.56	87	38	49	0.56	3.02E-09	1.23803E-09	*DICER1*	-	ENST00000343455	chr14:g.95574253C>T
3	Active	2	198266834	T	C	168	106	62	0.37	144	87	57	0.40	2.11E-15	5.81329E-16	*SF3B1*	-	ENST00000335508	chr2:g.198266834T>C
4	Active	1	22211932	G	C	86	68	18	0.21	156	152	4	0.03	NA	NA	*HSPG2*	-	ENST00000374695	chr1:g.22211932G>C
4	Active	6	100841696	C	T	53	41	12	0.23	91	88	3	0.03	NA	NA	*SIM1*	-	ENST00000369208	chr6:g.100841696C>T
4	Active	11	102207656	AC	A	39	38	1	0.00	24	16	8	0.03	NA	NA	*BIRC3*	+	ENST00000263464	chr11:g.102207657delC
4	Active	2	110936097	A	G	46	35	11	0.24	41	21	20	0.49	NA	NA	*NPHP1*	-	ENST00000316534	chr2:g.110936097A>G
4	Active	1	154544179	G	C	96	77	19	0.20	214	211	3	0.02	NA	NA	*CHRNB2*	+	ENST00000368476	chr1:g.154544179G>C
7	Active	19	2917842	G	A	126	96	30	0.24	175	122	53	0.30	NA	NA	*ZNF57*	+	ENST00000306908	chr19:g.2917842G>A
7	Active	1	19412654	G	C	95	79	16	0.16	101	59	42	0.42	NA	NA	*UBR4*	-	ENST00000375254	chr1:g.19412654G>C
7	Active	14	23863422	G	A	67	63	4	0.06	62	45	17	0.27	NA	NA	*MYH6*	-	ENST00000405093	chr14:g.23863422G>A
7	Active	3	38182641	T	C	79	65	14	0.18	85	53	32	0.38	NA	NA	*MYD88*	+	ENST00000396334	chr3:g.38182641T>C
7	Active	22	43524549	T	G	85	85	0	0.00	113	78	35	0.31	NA	NA	*BIK*	+	ENST00000216115	chr22:g.43524549T>G
7	Active	11	61110079	C	T	86	75	11	0.13	93	59	34	0.37	NA	NA	*DAK*	+	ENST00000394900	chr11:g.61110079C>T
7	Active	10	114920422	C	T	35	28	7	0.20	44	44	0	0.00	NA	NA	*TCF7L2*	+	ENST00000543371	chr10:g.114920422C>T
7	Active	3	173998572	C	T	71	70	1	0.01	46	30	16	0.35	NA	NA	*NLGN1*	+	ENST00000457714	chr3:g.173998572C>T
7	Active	1	210536196	A	T	60	48	12	0.20	55	55	0	0.00	NA	NA	*HHAT*	+	ENST00000545154	chr1:g.210536196A>T
8	Active	17	1373945	G	A	94	78	16	0.16	56	44	12	0.21	1.91E-07	5.22E-08	*MYO1C*	-	ENST00000359786	chr17:g.1373945G>A
8	Active	17	3627347	C	T	42	36	6	0.14	41	29	12	0.29	0.166424406	0.005247744	*GSG2*	+	ENST00000325418	chr17:g.3627347C>T
8	Active	19	7584939	G	T	135	62	73	0.54	52	26	26	0.50	0.004598701	0.030452023	*ZNF358*	+	ENST00000597229	chr19:g.7584939G>T
8	Active	16	20422874	A	G	171	87	84	0.49	125	62	63	0.50	8.10E-23	8.64E-21	*ACSM5*	+	ENST00000331849	chr16:g.20422874A>G
8	Active	12	54396320	G	T	72	37	35	0.49	90	39	51	0.57	0.005326196	0.000509115	*HOXC9*	+	ENST00000303450	chr12:g.54396320G>T
8	Active	7	128037043	C	T	60	34	26	0.43	45	28	17	0.38	9.25E-05	0.001643909	*IMPDH1*	-	ENST00000338791	chr7:g.128037043C>T
8	Active	2	238289980	G	C	56	29	27	0.48	44	23	21	0.48	0.000235004	0.000463391	*COL6A3*	-	ENST00000295550	chr2:g.238289980G>C
8	Active	2	238289984	C	G	53	28	25	0.47	44	23	21	0.48	2.74E-05	6.57E-05	*COL6A3*	-	ENST00000295550	chr2:g.238289984C>G
8	Active	1	247876023	AAGTCCTGGGGC	A	135	88	47	0.39	120	75	45	0.47	1.85E-16	2.48E-19	*OR6F1*	-	ENST00000302084	chr1:g.247876026_247876036delTCCTGGGGCAG
10	Active	12	7469772	T	G	10	9	1	0.10	26	17	9	0.35	0.25045045	3.24E-05	*ACSM4*	+	ENST00000399422	chr12:g.7469772T>G
10	Active	4	13476013	G	A	44	18	26	0.64	51	32	19	0.37	8.15E-07	0.004844448	*RAB28*	-	ENST00000330852	chr4:g.13476013G>A
10	Active	X	41206178	TTGAAGCTAAACAAGAAG	T	10	10	0	0.00	26	10	16	0.65	1	3.0737E-09	*DDX3X*	+	ENST00000399959	chrX:g.41206181_41206197delAAGCTAAACAAGAAGTG
10	Active	6	41903704	T	C	16	16	0	0.00	107	73	34	0.32	1	3.66E-05	*CCND3*	-	ENST00000372991	chr6:g.41903704T>C
10	Active	1	85790509	C	G	26	25	1	0.04	72	44	28	0.39	1	1.59E-06	*DDAH1*	-	ENST00000284031	chr1:g.85790509C>G
10	Active	2	102486822	T	C	16	14	2	0.13	62	37	25	0.42	0.053763441	2.49E-08	*MAP4K4*	+	ENST00000347699	chr2:g.102486822T>C
11	Active	7	2983910	C	T	35	26	9	0.26	46	20	26	0.57	0.432554191	2.98814E-05	*CARD11*	-	ENST00000396946	chr7:g.2983910C>T
11	Active	11	4471116	C	T	87	62	25	0.29	112	58	54	0.48	0.041186278	2.54E-06	*OR52K2*	+	ENST00000325719	chr11:g.4471116C>T
11	Active	4	5578105	G	A	92	67	25	0.26	131	67	64	0.48	0.223721672	1.06E-07	*EVC2*	-	ENST00000344408	chr4:g.5578105G>A
11	Active	7	13935577	G	T	41	39	2	0.05	45	25	20	0.44	1	0.005253295	*ETV1*	-	ENST00000430479	chr7:g.13935577G>T
11	Active	19	14208394	G	T	117	117	0	0.00	143	88	55	0.38	1	1.72E-07	*PRKACA*	-	ENST00000308677	chr19:g.14208394G>T
11	Active	8	17868817	A	C	132	61	71	0.54	98	52	46	0.47	3.35E-09	5.19E-07	*PCM1*	+	ENST00000325083	chr8:g.17868817A>C
11	Active	2	39477824	G	A	103	91	12	0.12	114	87	27	0.24	1	0.030746752	*MAP4K3*	-	ENST00000263881	chr2:g.39477824G>A
11	Active	2	47184026	G	A	68	47	21	0.31	83	58	25	0.30	1.81E-05	8.52E-06	*TTC7A*	+	ENST00000319190	chr2:g.47184026G>A
11	Active	15	52338664	G	C	110	58	52	0.47	92	56	36	0.39	0.000793197	0.025260554	*MAPK6*	+	ENST00000261845	chr15:g.52338664G>C
11	Active	3	57143586	G	T	35	29	6	0.15	39	23	16	0.59	0.746790379	3.30E-06	*IL17RD*	-	ENST00000296318	chr3:g.57143586G>T
11	Active	12	59280632	T	C	90	41	49	0.54	74	35	39	0.53	3.24E-05	0.00014303	*LRIG3*	-	ENST00000320743	chr12:g.59280632T>C
11	Active	6	75844556	G	A	72	72	0	0.00	79	39	40	0.51	1	0.001944378	*COL12A1*	-	ENST00000322507	chr6:g.75844556G>A
11	Active	15	79051848	GTGGGCAGC	G	70	46	24	0.43	76	48	28	0.44	1.72E-10	1.57E-11	*ADAMTS7*	-	ENST00000388820	chr15:g.79051852_79051859delGCAGCTGG
11	Active	16	88695259	C	T	89	62	27	0.30	142	64	78	0.55	0.356503224	4.61062E-08	*ZC3H18*	+	ENST00000301011	chr16:g.88695259C>T
11	Active	12	90020353	AATGGCTGCATTTCC	A	105	91	14	0.13	94	71	23	0.28	0.001255532	2.06E-07	*ATP2B1*	-	ENST00000428670	chr12:g.90020363_90020376delTTTCCATGGCTGCA
11	Active	12	109629466	G	T	95	50	45	0.47	111	55	56	0.50	0.000510416	0.000109252	*ACACB*	+	ENST00000338432	chr12:g.109629466G>T
11	Active	4	155161841	C	A	92	74	18	0.19	115	66	49	0.43	0.132755767	8.63E-07	*DCHS2*	-	ENST00000357232	chr4:g.155161841C>A
11	Active	1	179833929	C	T	124	71	53	0.43	110	65	45	0.41	0.005193724	0.011752389	*IFRG15*	-	ENST00000553856	chr1:g.179833929C>T
11	Active	2	189918658	C	T	107	81	26	0.24	110	52	58	0.53	0.328771722	1.47E-07	*COL5A2*	-	ENST00000374866	chr2:g.189918658C>T
11	Active	2	198266822	T	A	167	117	50	0.30	146	83	63	0.43	0.139540775	0.000106203	*SF3B1*	-	ENST00000335508	chr2:g.198266822T>A
12	Active	10	69366777	G	T	91	88	3	0.03	54	44	10	0.19	NA	NA	*CTNNA3*	-	ENST00000433211	chr10:g.69366777G>T
12	Active	7	124532330	G	C	21	14	7	0.33	18	15	3	0.17	NA	NA	*POT1*	-	ENST00000357628	chr7:g.124532330G>C
12	Active	9	139390966	G	A	76	40	36	0.47	90	54	36	0.40	NA	NA	*NOTCH1*	-	ENST00000277541	chr9:g.139390966G>A
12	Active	1	144912191	G	T	109	83	26	0.25	80	62	18	0.23	NA	NA	*PDE4DIP*	-	ENST00000369356	chr1:g.144912191G>T
14	Active	19	11917219	C	T	80	61	19	0.24	155	97	58	0.37	NA	NA	*ZNF491*	+	ENST00000323169	chr19:g.11917219C>T
14	Active	16	14014066	TGCTGGAGTACGA	T	33	26	7	0.19	181	126	55	0.36	NA	NA	*ERCC4*	+	ENST00000311895	chr16:g.14014069_14014080delTGGAGTACGAGC
14	Active	8	36675233	G	A	49	39	10	0.20	101	57	44	0.44	NA	NA	*KCNU1*	+	ENST00000399881	chr8:g.36675233G>A
14	Active	10	46965747	T	C	101	82	19	0.19	287	215	72	0.24	NA	NA	*SYT15*	-	ENST00000374321	chr10:g.46965747T>C
14	Active	1	109544932	T	C	70	55	15	0.21	117	81	36	0.31	NA	NA	*WDR47*	-	ENST00000400794	chr1:g.109544932T>C
14	Active	1	144881463	C	T	103	79	24	0.23	188	141	47	0.25	NA	NA	*PDE4DIP*	-	ENST00000369356	chr1:g.144881463C>T
15	Active	17	7579369	G	C	88	79	9	0.10	79	61	18	0.23	NA	NA	*TP53*	-	ENST00000269305	chr17:g.7579369G>C
15	Active	17	15207297	G	A	46	37	9	0.20	55	45	10	0.18	NA	NA	*TEKT3*	-	ENST00000395930	chr17:g.15207297G>A
15	Active	9	139390648	CAG	C	118	77	41	0.38	123	76	47	0.43	NA	NA	*NOTCH1*	-	ENST00000277541	chr9:g.139390649_139390650delAG
15	Active	5	177633767	G	C	105	80	25	0.24	122	86	36	0.30	NA	NA	*HNRNPAB*	+	ENST00000358344	chr5:g.177633767G>C
15	Active	2	198266709	C	G	81	60	21	0.26	97	84	13	0.14	NA	NA	*SF3B1*	-	ENST00000335508	chr2:g.198266709C>G
15	Active	1	245848983	C	T	182	164	18	0.10	212	176	36	0.18	NA	NA	*KIF26B*	+	ENST00000407071	chr1:g.245848983C>T
17	Active	1	3397035	C	T	129	88	41	0.32	115	60	55	0.48	1.00E-07	2.29E-14	*ARHGEF16*	+	ENST00000378378	chr1:g.3397035C>T
17	Active	11	5536476	G	C	53	24	29	0.55	43	22	21	0.49	0.000103489	0.000535651	*UBQLNL*	-	ENST00000380184	chr11:g.5536476G>C
17	Active	19	6586225	C	T	306	222	84	0.27	266	140	126	0.46	3.16E-10	4.48E-23	*CD70*	-	ENST00000245903	chr19:g.6586225C>T
17	Active	10	8111467	C	T	50	36	14	0.29	39	24	15	0.39	0.027091954	0.002772358	*GATA3*	+	ENST00000379328	chr10:g.8111467C>T
17	Active	12	38714351	T	G	121	59	62	0.51	91	42	49	0.54	0.000301705	0.000190807	*ALG10B*	+	ENST00000308742	chr12:g.38714351T>G
17	Active	20	49620602	A	G	321	223	98	0.28	290	149	141	0.47	7.46E-14	1.86E-27	*KCNG1*	-	ENST00000371571	chr20:g.49620602A>G
17	Active	6	76728526	C	T	52	32	20	0.38	48	24	24	0.50	9.60E-06	4.22E-08	*IMPG1*	-	ENST00000369950	chr6:g.76728526C>T
17	Active	16	88791902	C	T	94	69	25	0.27	73	37	36	0.49	0.00039571	2.05E-09	*PIEZO1*	-	ENST00000301015	chr16:g.88791902C>T
17	Active	6	114281146	G	A	59	49	10	0.17	64	32	32	0.50	0.124557224	2.45813E-06	*HDAC2*	-	ENST00000519065	chr6:g.114281146G>A
17	Active	3	123167134	C	A	23	20	3	0.13	29	16	13	0.45	0.389864893	4.74E-05	*ADCY5*	-	ENST00000462833	chr3:g.123167134C>A
17	Active	4	140616396	G	T	56	19	37	0.67	60	38	22	0.37	4.71E-05	0.028585477	*MGST2*	+	ENST00000265498	chr4:g.140616396G>T
17	Active	9	140941381	G	A	190	131	59	0.31	161	74	87	0.54	1.08E-15	6.02E-32	*CACNA1B*	+	ENST00000371355	chr9:g.140941381G>A
17	Active	1	156941539	G	A	273	191	82	0.33	248	130	118	0.47	1.09E-14	3.74E-25	*ARHGEF11*	-	ENST00000368194	chr1:g.156941539G>A
17	Active	3	197500300	G	A	78	58	20	0.34	63	43	20	0.32	1.95E-07	3.13E-06	*FYTTD1*	+	ENST00000241502	chr3:g.197500300G>A
23	Active	17	1782620	G	A	130	91	39	0.30	91	78	13	0.14	1.45E-05	0.04790185	*RPA1*	+	ENST00000254719	chr17:g.1782620G>A
23	Active	2	17962005	C	G	156	77	79	0.50	138	70	68	0.49	1.91E-08	3.97E-08	*GEN1*	+	ENST00000381254	chr2:g.17962005C>G
23	Active	15	59515322	AATACCCGCC	A	95	62	33	0.41	70	40	30	0.51	1.82E-11	1.12E-13	*MYO1E*	-	ENST00000288235	chr15:g.59515324_59515332delTACCCGCCA
23	Active	7	105135664	C	T	117	58	59	0.50	138	80	58	0.42	0.000563071	0.005729271	*PUS7*	-	ENST00000356362	chr7:g.105135664C>T
24	Active	11	10776609	G	T	101	63	38	0.38	94	46	48	0.51	7.84E-07	1.78E-09	*CTR9*	+	ENST00000361367	chr11:g.10776609G>T

24	Active	11	17793719	G	A	69	30	39	0.56	56	24	32	0.57	1.21E-10	2.50E-10	*KCNC1*	+	ENST00000265969	chr11:g.17793719G>A
24	Active	8	19681436	G	T	94	47	47	0.50	111	46	65	0.59	5.84E-05	5.65E-07	*INTS10*	+	ENST00000397977	chr8:g.19681436G>T
24	Active	13	26273377	G	A	80	46	34	0.43	73	34	39	0.53	0.000193396	2.56E-06	*ATP8A2*	+	ENST00000381655	chr13:g.26273377G>A
24	Active	16	66919665	C	G	21	16	5	0.24	28	14	14	0.50	0.132874912	0.002720275	*PDP2*	+	ENST00000311765	chr16:g.66919665C>G
24	Active	X	70339253	G	C	68	53	15	0.22	59	47	12	0.20	0.00013012	0.000395992	*MED12*	+	ENST00000374080	chrX:g.70339253G>C
24	Active	9	77684922	G	T	93	47	46	0.49	85	43	42	0.46	4.94E-12	1.58E-10	*NMRK1*	-	ENST00000361092	chr9:g.77684922G>T
24	Active	9	78749075	G	A	96	50	46	0.48	85	43	42	0.49	2.87E-10	2.68E-10	*PCSK5*	+	ENST00000545128	chr9:g.78749075G>A
24	Active	9	88162066	T	G	127	65	62	0.51	138	70	68	0.49	7.71E-07	2.11E-06	*AGTPBP1*	-	ENST00000376083	chr9:g.88162066T>G
24	Active	11	102207675	GAAGA	G	43	40	3	0.03	56	40	16	0.29	NA	NA	*BIRC3*	+	ENST00000263464	chr11:g.102207681_102207684delAGAA
24	Active	5	133328007	T	G	76	44	32	0.41	64	30	34	0.53	4.67E-09	4.45E-12	*VDAC1*	-	ENST00000265333	chr5:g.133328007T>G
24	Active	X	140994765	T	G	536	284	252	0.47	199	97	102	0.51	1.27E-10	5.97E-12	*MAGEC1*	+	ENST00000285879	chrX:g.140994765T>G
24	Active	4	155720111	G	A	97	55	42	0.43	72	46	26	0.36	0.000134519	0.002426506	*RBM46*	+	ENST00000281722	chr4:g.155720111G>A
24	Active	3	179294488	G	A	154	79	75	0.49	134	68	66	0.49	5.94E-05	5.19E-05	*ACTL6A*	+	ENST00000429709	chr3:g.179294488G>A
24	Active	2	234669305	G	T	115	63	52	0.45	93	41	52	0.56	1.15E-11	2.21E-14	*UGT1A1*	+	ENST00000305208	chr2:g.234669305G>T
33	Active	9	23701530	T	C	97	91	6	0.06	92	73	19	0.21	0.177009226	0.000355176	*ELAVL2*	-	ENST00000397312	chr9:g.23701530T>C
33	Active	1	44877864	C	T	69	36	33	0.48	93	50	43	0.46	1.44E-07	1.46E-07	*RNF220*	+	ENST00000355387	chr1:g.44877864C>T
33	Active	17	54912191	C	T	18	13	5	0.28	34	15	19	0.56	0.12605785	0.001005219	*DGKE*	+	ENST00000284061	chr17:g.54912191C>T
33	Active	3	81586147	T	A	48	24	24	0.50	73	32	41	0.56	4.04E-10	3.94E-13	*GBE1*	-	ENST00000429644	chr3:g.81586147T>A
33	Active	9	98229620	T	A	17	10	7	0.41	34	13	21	0.62	7.36E-05	6.12714E-10	*PTCH1*	-	ENST00000331920	chr9:g.98229620T>A
33	Active	2	99797359	G	T	84	47	37	0.43	105	50	55	0.52	3.56E-06	6.63E-08	*MITD1*	-	ENST00000289359	chr2:g.99797359G>T
33	Active	5	151175120	G	GA	72	51	21	0.50	82	57	25	0.51	4.04E-10	9.45295E-11	*G3BP1*	+	ENST00000394123	chr5:g.151175121dupA
34	Active	4	7735097	C	T	73	50	23	0.31	81	40	41	0.49	1.86E-10	2.98E-18	*SORCS2*	+	ENST00000507866	chr4:g.7735097C>T
34	Active	8	25268713	G	A	105	53	52	0.50	118	55	63	0.52	6.36E-21	1.37E-22	*DOCK5*	+	ENST00000276440	chr8:g.25268713G>A
34	Active	X	27839906	G	T	54	6	48	0.88	60	0	60	1.00	5.54E-27	5.34E-37	*MAGEB10*	+	ENST00000356790	chrX:g.27839906G>T
34	Active	13	31221142	C	T	54	51	3	0.06	55	42	13	0.25	0.064738001	3.57E-06	*USPL1*	+	ENST00000255304	chr13:g.31221142C>T
34	Active	6	44232738	TGTAA	T	23	20	3	0.13	48	44	4	0.83	2.00E-02	0.03770484	*NFKBIE*	-	ENST00000275015	chr6:g.44232741_44232744delAAGT
34	Active	19	54705126	T	G	104	54	50	0.50	126	60	66	0.52	1.36E-25	1.70E-29	*RPS9*	+	ENST00000302907	chr19:g.54705126T>G
34	Active	1	70486747	C	T	65	41	24	0.34	80	42	38	0.47	5.86E-11	1.68E-17	*LRRC7*	+	ENST00000035383	chr1:g.70486747C>T
34	Active	6	147067083	C	A	69	35	34	0.52	58	35	23	0.40	4.76E-13	2.65E-09	*ADGB*	+	ENST00000397944	chr6:g.147067083C>A
34	Active	2	160005758	C	A	110	54	56	0.50	109	50	59	0.54	4.30E-22	2.41E-24	*TANC1*	+	ENST00000263635	chr2:g.160005758C>A
34	Active	2	176982114	G	A	215	125	90	0.40	228	115	113	0.51	7.31E-29	3.00E-38	*HOXD10*	+	ENST00000249501	chr2:g.176982114G>A
34	Active	2	198267360	T	C	31	17	14	0.47	25	13	12	0.50	4.87E-07	5.49304E-07	*SF3B1*	-	ENST00000335508	chr2:g.198267360T>C
37	Active	19	2413725	G	A	54	32	22	0.41	60	30	30	0.50	8.29E-10	7.67E-13	*TMPRSS9*	+	ENST00000332578	chr19:g.2413725G>A
37	Active	17	10400691	T	C	81	48	33	0.41	64	34	30	0.47	9.83E-12	8.23E-13	*MYH1*	-	ENST00000226207	chr17:g.10400691T>C
37	Active	22	31487265	G	A	164	91	73	0.45	134	66	68	0.51	7.81E-27	1.82E-29	*SMTN*	+	ENST00000358743	chr22:g.31487265G>A
37	Active	3	38182259	T	C	72	38	34	0.47	47	22	25	0.53	2.53E-14	4.76373E-14	*MYD88*	+	ENST00000396334	chr3:g.38182259T>C
37	Active	4	46979479	G	A	51	33	18	0.35	48	25	23	0.48	2.18E-05	1.83E-07	*GABRA4*	-	ENST00000264318	chr4:g.46979479G>A
37	Active	5	52235686	A	C	55	25	30	0.55	66	37	29	0.44	8.41E-12	1.56E-09	*ITGA1*	+	ENST00000282588	chr5:g.52235686A>C
37	Active	7	53103839	C	T	107	58	49	0.46	111	59	52	0.47	3.40E-17	7.80E-18	*POM121L12*	+	ENST00000408890	chr7:g.53103839C>T
37	Active	18	56202479	C	A	93	48	45	0.48	81	54	27	0.33	1.33E-17	4.83E-11	*ALPK2*	-	ENST00000361673	chr18:g.56202479C>A
37	Active	17	57350183	G	T	63	38	25	0.40	76	41	35	0.46	1.83E-10	4.33E-13	*GDPD1*	+	ENST00000284116	chr17:g.57350183G>T
37	Active	18	61647131	G	T	93	52	41	0.44	103	52	51	0.50	1.50E-13	3.07E-16	*SERPINB8*	+	ENST00000397985	chr18:g.61647131G>T
37	Active	17	80285085	T	C	172	93	79	0.46	138	68	70	0.51	1.31E-32	9.20E-34	*SECTM1*	-	ENST00000269389	chr17:g.80285085T>C
37	Active	11	92498066	C	T	47	25	22	0.47	35	20	15	0.43	9.15E-08	1.14E-06	*FAT3*	+	ENST00000298047	chr11:g.92498066C>T
37	Active	3	99514127	A	G	375	187	188	0.51	304	161	143	0.44	2.69E-38	2.62E-32	*COL8A1*	+	ENST00000261037	chr3:g.99514127A>G
37	Active	9	110248108	G	C	48	28	20	0.42	39	18	21	0.54	2.11E-09	1.22E-11	*KLF4*	-	ENST00000374672	chr9:g.110248108G>C
37	Active	12	122359252	A	G	145	88	57	0.39	156	87	69	0.44	3.78E-23	1.25E-27	*WDR66*	+	ENST00000288912	chr12:g.122359252A>G
37	Active	X	141291746	G	A	72	2	70	0.97	52	1	51	0.98	1.01E-44	1.68E-38	*MAGEC2*	-	ENST00000247452	chrX:g.141291746G>A
38	Active	4	619750	G	A	66	43	23	0.35	114	82	32	0.28	5.41E-05	0.000181168	*PDE6B*	+	ENST00000496514	chr4:g.619750G>A
38	Active	1	934990	A	G	43	17	26	0.60	65	27	38	0.58	8.62E-07	2.11E-07	*HES4*	-	ENST00000428771	chr1:g.934990A>G
38	Active	12	4383309	G	A	114	87	27	0.24	161	107	54	0.34	1.84E-05	2.31569E-08	*CCND2*	+	ENST00000261254	chr12:g.4383309G>A
38	Active	4	7435201	C	T	122	96	26	0.21	200	153	47	0.24	0.041815224	0.025620471	*PSAPL1*	-	ENST00000319098	chr4:g.7435201C>T
38	Active	19	11411979	GA	G	27	19	8	0.27	34	16	18	0.53	0.072973903	0.000578143	*TSPAN16*	+	ENST00000316737	chr19:g.11411980delA
38	Active	8	17065563	A	G	75	54	21	0.28	92	73	19	0.21	6.55E-05	0.000835922	*ZDHHC2*	+	ENST00000262096	chr8:g.17065563A>G
38	Active	9	19372143	G	A	62	30	32	0.51	80	40	40	0.50	2.01E-07	1.54E-07	*DENND4C*	+	ENST00000380437	chr9:g.19372143G>A
38	Active	17	30648093	G	A	134	97	37	0.26	197	146	51	0.26	9.51E-10	2.35E-10	*RHBDL3*	+	ENST00000269051	chr17:g.30648093G>A
38	Active	19	38905715	G	C	15	12	3	0.20	34	21	13	0.38	NA	NA	*RASGRP4*	-	ENST00000587738	chr19:g.38905715G>C
38	Active	19	54646887	G	A	73	62	11	0.15	85	65	20	0.24	0.111721173	0.020624479	*CNOT3*	+	ENST00000406403	chr19:g.54646887G>A
38	Active	11	65348585	C	T	168	124	44	0.26	284	214	70	0.27	3.03E-12	7.20E-13	*EHBP1L1*	+	ENST00000309295	chr11:g.65348585C>T
38	Active	15	68583228	A	G	114	55	59	0.52	173	96	77	0.45	5.48E-06	3.50E-05	*FEM1B*	+	ENST00000306917	chr15:g.68583228A>G
38	Active	11	102207721	T	A	37	33	4	0.11	41	35	6	0.15	0.6414839	0.04052337	*BIRC3*	+	ENST00000263464	chr11:g.102207721T>A
38	Active	6	136599585	C	T	130	102	28	0.22	166	128	38	0.23	0.04410088	0.016163751	*BCLAF1*	-	ENST00000531224	chr6:g.136599585C>T
38	Active	9	139390648	CAG	C	154	111	43	0.47	212	146	66	0.50	0.001239387	0.000436421	*NOTCH1*	-	ENST00000277541	chr9:g.139390649_139390650delAG
38	Active	5	140736824	A	G	164	86	78	0.48	263	148	115	0.41	3.91E-07	4.63E-06	*PCDHGA4*	+	ENST00000571252	chr5:g.140736824A>G
38	Active	X	153678609	T	A	66	57	9	0.14	91	70	21	0.23	0.00010755	1.59729E-08	*FAM50A*	+	ENST00000393600	chrX:g.153678609T>A
38	Active	4	187527303	G	A	28	12	16	0.57	46	24	22	0.48	2.71E-08	8.55112E-08	*FAT1*	-	ENST00000441802	chr4:g.187527303G>A
39	Active	19	1440424	G	C	246	129	117	0.49	164	80	84	0.52	2.31E-36	7.60087E-37	*RPS15*	+	ENST00000586686	chr19:g.1440424G>C
39	Active	19	1785541	G	A	24	14	10	0.41	14	9	5	0.36	0.002461976	0.011782639	*ATP8B3*	-	ENST00000310127	chr19:g.1785541G>A
39	Active	7	4901143	G	A	101	48	53	0.52	67	30	37	0.56	1.81E-20	3.40E-19	*PAPOLB*	-	ENST00000404991	chr7:g.4901143G>A
39	Active	17	11725771	C	T	38	16	22	0.58	49	20	29	0.58	1.92E-09	2.37E-10	*DNAH9*	+	ENST00000262442	chr17:g.11725771C>T
39	Active	17	15492503	ATTC	A	244	145	99	0.54	138	81	57	0.55	4.28E-35	2.02E-30	*CDRT1*	-	ENST00000395906	chr17:g.15492506_15492508delCTT
39	Active	1	20827823	C	T	279	129	150	0.57	202	106	96	0.48	8.85E-45	8.45E-36	*MUL1*	-	ENST00000264198	chr1:g.20827823C>T
39	Active	16	23403800	C	T	53	32	21	0.40	25	14	11	0.44	1.55E-09	6.46E-08	*COG7*	-	ENST00000307149	chr16:g.23403800C>T
39	Active	2	27521496	G	A	101	50	51	0.49	72	35	37	0.53	6.02E-19	2.83E-18	*TRIM54*	+	ENST00000296098	chr2:g.27521496G>A
39	Active	9	36171159	G	A	54	25	29	0.53	33	18	15	0.45	3.86E-10	2.37E-07	*CCIN*	+	ENST00000335119	chr9:g.36171159G>A
39	Active	19	48558213	C	A	15	7	8	0.57	16	11	5	0.33	7.78E-05	0.005982763	*PLA2G4C*	-	ENST00000599921	chr19:g.48558213C>A
39	Active	2	51255119	G	A	168	83	85	0.49	95	56	39	0.41	6.07E-29	1.67E-19	*NRXN1*	-	ENST00000404971	chr2:g.51255119G>A
39	Active	19	51507039	C	T	79	43	36	0.45	56	26	30	0.53	3.83E-15	6.87E-16	*KLK9*	-	ENST00000376832	chr19:g.51507039C>T
39	Active	12	51510124	C	T	70	31	39	0.56	55	26	29	0.54	1.38E-17	4.71E-15	*TFCP2*	-	ENST00000257915	chr12:g.51510124C>T
39	Active	20	57568692	A	C	150	70	80	0.54	116	56	60	0.52	3.45E-25	2.70E-22	*TH1L*	+	ENST00000344018	chr20:g.57568692A>C
39	Active	2	62067550	C	T	115	67	48	0.41	86	37	49	0.57	1.82E-14	8.72E-20	*FAM161A*	-	ENST00000404929	chr2:g.62067550C>T
39	Active	16	67702345	C	G	94	53	41	0.45	64	31	33	0.51	4.02E-16	1.97E-16	*C16orf86*	+	ENST00000403458	chr16:g.67702345C>G
39	Active	16	77465256	A	G	81	42	39	0.49	54	32	22	0.41	1.30E-15	3.62E-11	*ADAMTS18*	-	ENST00000282849	chr16:g.77465256A>G
39	Active	1	78428614	TC	T	82	59	23	0.51	54	36	18	0.54	7.71E-07	7.38219E-07	*FUBP1*	-	ENST00000370768	chr1:g.78428616delC
39	Active	7	87022368	A	G	55	29	26	0.48	57	20	37	0.65	1.17E-08	6.45E-13	*CROT*	+	ENST00000419147	chr7:g.87022368A>G
39	Active	14	88651982	G	A	47	22	25	0.53	33	18	15	0.45	1.09E-10	7.00E-08	*KCNK10*	-	ENST00000319231	chr14:g.88651982G>A
39	Active	3	98519531	G	A	103	58	45	0.43	69	41	28	0.38	3.61E-15	1.31E-11	*DCBLD2*	-	ENST00000326840	chr3:g.98519531G>A
39	Active	8	144660093	C	T	116	57	59	0.51	78	34	44	0.57	5.53E-23	1.41E-22	*NAPRT1*	-	ENST00000449291	chr8:g.144660093C>T
39	Active	1	182993152	C	T	103	51	52	0.50	68	41	27	0.40	1.09E-17	6.07E-12	*LAMC1*	+	ENST00000258341	chr1:g.182993152C>T
40	Active	17	3030338	C	T	135	89	46	0.34	156	75	81	0.52	9.30E-16	5.24E-27	*OR1G1*	-	ENST00000328890	chr17:g.3030338C>T
40	Active	12	4409156	T	A	261	180	81	0.29	242	138	104	0.45	5.44E-20	3.35977E-33	*CCND2*	+	ENST00000261254	chr12:g.4409156T>A
40	Active	19	10257155	T	A	72	50	22	0.31	82	45	37	0.44	5.37E-09	6.47E-14	*DNMT1*	-	ENST00000359526	chr19:g.10257155T>A
40	Active	18	24442393	G	A	29	19	10	0.34	31	15	16	0.52	0.000767735	3.02E-06	*AQP4*	-	ENST00000383168	chr18:g.24442393G>A
40	Active	11	64604226	G	A	70	48	22	0.31	83	49	34	0.41	8.95E-09	4.52E-12	*CDC42BPG*	-	ENST00000342711	chr11:g.64604226G>A
40	Active	8	113299430	C	T	42	30	12	0.29	54	33	21	0.38	7.23E-05	9.30E-07	*CSMD3*	-	ENST00000297405	chr8:g.113299430C>T
40	Active	4	134073244	C	T	26	17	9	0.35	24	17	7	0.33	0.002988528	0.008620253	*PCDH10*	+	ENST00000264360	chr4:g.134073244C>T

40	Active	9	135917685	A	G	109	74	35	0.32	121	59	62	0.52	1.46E-13	3.24E-24	*GTF3C5*	+	ENST00000372108	chr9:g.135917685A>G
40	Active	5	140744589	C	T	35	22	13	0.37	47	34	13	0.28	9.67E-06	0.000116671	*PCDHGA5*	+	ENST00000518069	chr5:g.140744589C>T
43	Active	11	5010947	CT	C	47	42	5	0.07	36	24	12	0.36	0.548531106	0.001967917	*MMP26*	+	ENST00000380390	chr11:g.5010948delT
43	Active	13	22084153	C	T	26	20	6	0.23	22	11	11	0.48	0.156711805	0.011768918	*EFHA1*	-	ENST00000382374	chr13:g.22084153C>T
43	Active	1	27100919	C	T	114	106	8	0.07	110	70	40	0.36	0.110831781	4.69701E-07	*ARID1A*	+	ENST00000324856	chr1:g.27100919C>T
43	Active	6	28333223	A	T	31	30	1	0.03	35	19	16	0.47	1	0.007381863	*ZKSCAN3*	+	ENST00000377255	chr6:g.28333223A>T
43	Active	18	33783144	G	A	106	104	2	0.02	90	58	32	0.35	0.524275806	3.26E-09	*MOCOS*	+	ENST00000261326	chr18:g.33783144G>A
43	Active	6	44232738	TGTAA	T	25	20	5	0.20	20	9	11	0.45	1	0.009834369	*NFKBIE*	-	ENST00000275015	chr6:g.44232741_44232744delAAGT
43	Active	19	45579465	ACCT	A	11	6	5	0.60	32	19	13	0.50	0.033841159	0.030428814	*ZNF296*	-	ENST00000303809	chr19:g.45579469_45579471delCCT
43	Active	3	46497459	G	T	68	63	5	0.07	60	34	26	0.45	0.661876382	3.90E-06	*LTF*	-	ENST00000231751	chr3:g.46497459G>T
43	Active	19	55623922	C	T	73	62	11	0.15	70	27	43	0.62	0.111721173	3.63E-07	*PPP1R12C*	-	ENST00000263433	chr19:g.55623922C>T
43	Active	10	99327742	C	T	122	101	21	0.17	95	59	36	0.39	3.20E-06	1.62E-13	*UBTD1*	+	ENST00000370664	chr10:g.99327742C>T
43	Active	3	119962595	A	T	40	35	5	0.13	29	17	12	0.41	0.022663051	3.38E-06	*GPR156*	-	ENST00000464295	chr3:g.119962595A>T
43	Active	X	153678605	C	G	51	41	10	0.20	32	0	32	1.00	0.01140667	1.69E-17	*FAM50A*	+	ENST00000393600	chrX:g.153678605C>G
43	Active	2	160289402	T	C	60	55	5	0.08	51	26	25	0.49	0.329265671	4.66E-05	*BAZ2B*	-	ENST00000392783	chr2:g.160289402T>C
43	Active	2	189906352	G	A	83	73	10	0.12	90	43	47	0.52	0.002291751	5.99E-15	*COL5A2*	-	ENST00000374866	chr2:g.189906352G>A
43	Active	1	215972294	C	A	101	91	10	0.10	82	36	46	0.56	0.00538123	9.81E-18	*USH2A*	-	ENST00000307340	chr1:g.215972294C>A
43	Active	1	231337126	G	A	135	113	22	0.16	82	54	28	0.34	0.000144371	4.60E-09	*TRIM67*	+	ENST00000366653	chr1:g.231337126G>A

Abbreviations:
TP - time-point
TDS - targeted deep sequencing

cDNA change	Protein change	Exon	Variant classification	Type of mutation	Validated by TDS	Pfam domain	Cadd phred	Driver gene	Gene role	Is in cluster	Is in delicate domain	Mutation location	Driver mut prediction	Driver (known/predicted)	Driver statement
c.2528A>G	p.H843R	14	Missense	Passenger	NA		0.002					before_last_exon	passenger		predicted passenger
c.4235T>G	p.L1412R	36	Missense	Passenger	NA		16.38		LoF			before_last_exon	passenger		predicted passenger
c.1197C>A	p.S399R	11	Missense	Passenger	NA		24.3					before_last_exon	passenger		predicted passenger
c.5933_5941delAGTTTAAGG	p.E1978_K1980delEFK	35	InFrameDeletion	Passenger	NA	VWA	20.8					before_last_exon	passenger		predicted passenger
c.2200C>T	p.Q734*	11	Nonsense	Passenger	NA		40					before_last_exon	passenger		predicted passenger
c.1156G>C	p.G386R	9	Missense	Passenger	NA		17.1					before_last_portion	passenger		predicted passenger
c.1561G>C	p.E521Q	5	Missense	Passenger	NA		20.3					before_last_exon	passenger		predicted passenger
c.1150G>A	p.E384K	8	Missense	Passenger	NA		15.64					before_last_exon	passenger		predicted passenger
c.691A>G	p.K231E	1	Missense	Driver	No	RRM_1	26.1	other_tumor_driver	ambiguous			before_last_portion	TIER 2	predicted	predicted driver: tier 2
c.482C>G	p.P161R	2	Missense	Passenger	NA		0.001					before_last_portion	passenger		predicted passenger
c.775G>C	p.V259L	6	Missense	Passenger	NA		13.59					before_last_exon	passenger		predicted passenger
c.418G>A	p.A140T	5	Missense	Passenger	NA		23.8					before_last_exon	passenger		predicted passenger
c.1986C>G	p.H662Q	14	Missense	Driver	Yes		25.2	tumor_driver	Act			before_last_exon	TIER 1	predicted	predicted driver: tier 1
c.155T>C	p.L52P	1	Missense	Passenger	NA		23.7					before_last_exon	passenger		predicted passenger
c.50A>G	p.N17S	1	Missense	Passenger	NA	Ssu72	24.5					before_last_exon	passenger		predicted passenger
c.1723G>T	p.D575Y	13	Missense	Passenger	NA	TSP_1	35					before_last_exon	passenger		predicted passenger
c.89C>T	p.P30L	2	Missense	Passenger	NA		10.32					before_last_exon	passenger		predicted passenger
c.247T>A	p.S83T	3	Missense	Passenger	NA	Neurexophilin	13.78					before_last_portion	passenger		predicted passenger
c.307A>T	p.I103F	3	Missense	Passenger	NA	Secretogranin_V	25.5					before_last_exon	passenger		predicted passenger
c.625G>T	p.D209Y	4	Missense	Driver	Yes		26	other_tumor_driver	ambiguous			before_last_exon	TIER 2	predicted	predicted driver: tier 2
c.3151G>T	p.D1051Y	17	Missense	Passenger	NA	Calx-beta	26					before_last_exon	passenger		predicted passenger
c.3191A>C	p.E1064A	17	Missense	Passenger	NA	Calx-beta	8.036					before_last_exon	passenger		predicted passenger
c.771G>T	p.L257F	4	Missense	Passenger	NA		25.3					before_last_exon	passenger		predicted passenger
c.617G>A	p.R206H	7	Missense	Passenger	NA		35		Act			before_last_exon	passenger		predicted passenger
c.922A>G	p.I308V	9	Missense	Passenger	NA		22.5					before_last_exon	passenger		predicted passenger
c.1385G>T	p.G462V	7	Missense	Passenger	NA	DDE_Tnp_1_7	26.2					before_last_portion	passenger		predicted passenger
c.55T>C	p.C19R	1	Missense	Passenger	NA		25					before_last_exon	passenger		predicted passenger
c.112T>G	p.W38G	1	Missense	Passenger	NA		23					before_last_exon	passenger		predicted passenger
c.6205A>T	p.R2069W	11	Missense	Passenger	NA		23.4					before_last_portion	passenger		predicted passenger
c.2666G>C	p.G889A	16	Missense	Passenger	NA		23.2		Act			before_last_exon	passenger		predicted passenger
c.1603C>G	p.R535G	12	Missense	Passenger	NA	Vinculin	23					before_last_exon	passenger		predicted passenger
c.103C>A	p.L35M	1	Missense	Passenger	NA		14.73					before_last_portion	passenger		predicted passenger
c.1183G>A	p.A395T	7	Missense	Passenger	NA		3.566					before_last_exon	passenger		predicted passenger
c.566A>G	p.Y189C	10	Missense	Passenger	NA	Mtc	16.19					before_last_exon	passenger		predicted passenger
c.2071A>G	p.T691A	5	Missense	Passenger	NA		0.021					before_last_exon	passenger		predicted passenger
c.634C>T	p.Q212*	4	Nonsense	Passenger	NA		36		LoF			before_last_exon	passenger		predicted passenger
c.3737T>C	p.M1246T	15	Missense	Passenger	NA	SNF2_N	26					before_last_exon	passenger		predicted passenger
c.106G>A	p.E36K	1	Missense	Driver	No	Cyclin_N	29.4	other_tumor_driver	Act			before_last_exon	TIER 2	predicted	predicted driver: tier 2
c.125T>G	p.V42G	1	Missense	Driver	Yes	Cyclin_N	23.2	tumor_driver	Act			before_last_exon	TIER 2	predicted	predicted driver: tier 2
c.714A>C	p.L238F	3	Missense	Passenger	NA	7tm_1	23.6					before_last_exon	passenger		predicted passenger
c.5447C>T	p.A1816V	5	Missense	Passenger	NA		18.87					before_last_exon	passenger		predicted passenger
c.2061T>G	p.H687Q	15	Missense	Passenger	NA		9.484					before_last_exon	passenger		predicted passenger
c.1765C>T	p.P589S	10	Missense	Passenger	NA	EphA2_TM	25.7					before_last_exon	passenger		predicted passenger
c.1571G>A	p.R524Q	17	Missense	Passenger	NA		28.7					before_last_exon	passenger		predicted passenger
c.1633G>T	p.A545S	3	Missense	Passenger	NA		0.001					before_last_portion	passenger		predicted passenger
c.6194G>T	p.S2065I	43	Missense	Passenger	NA	Spectrin	22.9					before_last_exon	passenger		predicted passenger
c.7235G>A	p.G2412E	12	Missense	Passenger	NA	DND1_DSRM	23					last_portion	passenger		predicted passenger
c.248T>C	p.V83A	3	Missense	Passenger	NA		11.47					before_last_exon	passenger		predicted passenger
c.483C>A	p.S161R	5	Missense	Passenger	NA		12.14					before_last_portion	passenger		predicted passenger
c.1781C>T	p.P594L	1	Missense	Passenger	NA		24.3					before_last_exon	passenger		predicted passenger
c.746C>T	p.S249L	6	Missense	Passenger	NA	Sulfotransfer_1	22		Act			before_last_exon	passenger		predicted passenger
c.2081C>T	p.A694V	21	Missense	Passenger	NA		34					before_last_exon	passenger		predicted passenger
c.377G>A	p.R126H	4	Missense	Passenger	NA	RhoGAP	35					before_last_exon	passenger		predicted passenger
c.9062G>A	p.R3021Q	37	Missense	Passenger	NA	Pkinase	34					before_last_exon	passenger		predicted passenger
c.2994A>C	p.Q998H	21	Missense	Passenger	NA		10.9					before_last_exon	passenger		predicted passenger
c.287delA	p.E96Gfs*10	5	Frameshift	Passenger	NA	LAG1-DNAbind	34					before_last_exon	passenger		predicted passenger
c.1111C>T	p.P371S	6	Missense	Passenger	NA	zf-H2C2_2	16.61					before_last_portion	passenger		predicted passenger
c.367C>T	p.R123C	3	Missense	Passenger	NA	Trypsin	23.3					before_last_exon	passenger		predicted passenger
c.371C>T	p.T124M	2	Missense	Passenger	NA	Glyco_transf_8	32					before_last_exon	passenger		predicted passenger
c.2524G>A	p.G842R	8	Missense	Passenger	NA	DUF3513	34					before_last_portion	passenger		predicted passenger
c.1826C>G	p.P609R	2	Missense	Passenger	NA		25.2					before_last_exon	passenger		predicted passenger
c.12974A>G	p.D4325G	7	Missense	Passenger	NA		18					before_last_exon	passenger		predicted passenger
c.2845G>A	p.D949N	18	Missense	Passenger	NA		26.4					before_last_exon	passenger		predicted passenger
c.26T>G	p.L9R	2	Missense	Passenger	NA	AF-4	24	other_tumor_driver	Act			before_last_exon	passenger		predicted passenger
c.1909delT	p.C637Afs*15	2	Frameshift	Passenger	NA		24.9					before_last_portion	passenger		predicted passenger
c.890G>A	p.C297Y	8	Missense	Driver	Yes	Iso_dh	34	tumor_driver	Act			before_last_exon	TIER 1	predicted	predicted driver: tier 1
c.2251G>A	p.A751T	3	Missense	Passenger	NA		0.001					before_last_exon	passenger		predicted passenger
c.1561G>C	p.E521Q	11	Missense	Passenger	NA		11.35					before_last_portion	passenger		predicted passenger
c.4487T>C	p.M1496T	25	Missense	Passenger	NA		22.8					before_last_portion	passenger		predicted passenger
c.7117A>G	p.S2373G	2	Missense	Passenger	NA		0.005	other_tumor_driver	ambiguous			before_last_exon	passenger		predicted passenger
c.1744G>T	p.E582*	11	Nonsense	Passenger	NA	zf-H2C2_2	43					before_last_portion	passenger		predicted passenger
c.631G>T	p.E211*	7	Nonsense	Passenger	NA		38					before_last_exon	passenger		predicted passenger
c.263T>C	p.I88T	4	Missense	Passenger	NA	MIP	20.2					before_last_exon	passenger		predicted passenger
c.1163A>C	p.Q388P	11	Missense	Passenger	NA		25.5					before_last_exon	passenger		predicted passenger
c.521C>T	p.P174L	4	Missense	Passenger	NA	p450	25.2					before_last_exon	passenger		predicted passenger
c.1265A>C	p.Q422P	11	Missense	Passenger	NA		0.001					before_last_portion	passenger		predicted passenger
c.3728G>C	p.R1243P	21	Missense	Passenger	NA		3.49					before_last_exon	passenger		predicted passenger
c.3719T>C	p.L1240P	21	Missense	Passenger	NA		1.26					before_last_exon	passenger		predicted passenger
c.5569C>T	p.R1857C	34	Missense	Passenger	NA		34		Act			before_last_exon	passenger		predicted passenger
c.7039A>T	p.T2347S	2	Missense	Passenger	NA		0.019	other_tumor_driver	ambiguous			before_last_exon	passenger		predicted passenger
c.1540G>A	p.V514M	19	Missense	Passenger	NA		25.7					before_last_exon	passenger		predicted passenger
c.1645C>T	p.R549C	12	Missense	Passenger	NA		22.7					before_last_exon	passenger		predicted passenger
c.404G>A	p.R135Q	3	Missense	Passenger	NA	ASC	18.28					before_last_exon	passenger		predicted passenger
c.664C>T	p.Q222*	4	Nonsense	Passenger	NA		35					before_last_exon	passenger		predicted passenger
c.1454A>G	p.N485S	11	Missense	Passenger	NA		0.001					before_last_exon	passenger		predicted passenger
c.881_884delTGGA	p.L294Sfs*18	8	Frameshift	Passenger	NA		35					before_last_exon	passenger		predicted passenger

c.888delT	p.A298Pfs*15	8	Frameshift	Passenger	NA		17.8					before_last_exon	passenger		predicted passenger
c.890_906delGGGCCTCCCCTTCCTAC	p.G297Afs*2	8	Frameshift	Passenger	NA		34					before_last_exon	passenger		predicted passenger
c.2890C>T	p.P964S	15	Missense	Passenger	NA		28.7					before_last_exon	passenger		predicted passenger
c.764T>C	p.V255A	3	Missense	Passenger	NA	zf-H2C2_2	26.3					before_last_exon	passenger		predicted passenger
c.1471C>T	p.Q491*	13	Nonsense	Passenger	NA		35					before_last_exon	passenger		predicted passenger
c.1190A>G	p.N397S	11	Missense	Passenger	NA	FAD-oxidase_C	19.09					before_last_exon	passenger		predicted passenger
c.571G>C	p.A191P	7	Missense	Driver	Yes		28.8	other_tumor_driver	LoF			before_last_exon	TIER 2	predicted	predicted driver: tier 2
c.932C>G	p.T311S	10	Missense	Driver	Yes	CDC73_C	29.9	other_tumor_driver	LoF			before_last_exon	TIER 2	predicted	predicted driver: tier 2
c.1997A>C	p.K666T	14	Missense	Driver	Yes		27.6	tumor_driver	Act	in_cluster		before_last_exon	TIER 1	known	known in: AML
c.1000A>C	p.S334R	6	Missense	Passenger	NA	Raftlin	23.1					before_last_exon	passenger		predicted passenger
c.49_50delAC	p.T17Vfs*390	1	Frameshift	Passenger	NA		23.4		LoF			before_last_portion	passenger		predicted passenger
c.430C>T	p.H137Y	4	Missense	Driver	Yes		23	other_tumor_driver		in_cluster		before_last_portion	TIER 2	predicted	predicted driver: tier 2
c.221A>C	p.D74A	2	Missense	Driver	No	Ank_2	28.2	other_tumor_driver	LoF			before_last_exon	TIER 2	predicted	predicted driver: tier 2
c.1394T>C	p.L465P	3	Missense	Passenger	NA		0.009					before_last_exon	passenger		predicted passenger
c.176T>G	p.V59G	1	Missense	Passenger	NA	tRNA-synt_2c	25.7					before_last_exon	passenger		predicted passenger
c.1347T>G	p.S449R	13	Missense	Driver	Yes	DEAD	27.7	other_tumor_driver	Act			before_last_exon	TIER 2	predicted	predicted driver: tier 2
c.391C>T	p.P131S	4	Missense	Driver	Yes		24.1	other_tumor_driver		in_cluster		before_last_portion	TIER 2	predicted	predicted driver: tier 2
c.2365G>A	p.E789K	20	Missense	Passenger	NA		33					before_last_exon	passenger		predicted passenger
c.1027A>G	p.T343A	4	Missense	Passenger	NA		5.758					before_last_portion	passenger		predicted passenger
c.1033A>G	p.K345E	4	Missense	Passenger	NA		0.002					before_last_portion	passenger		predicted passenger
c.1594G>A	p.G532S	6	Missense	Passenger	NA		22.6					before_last_exon	passenger		predicted passenger
c.785C>T	p.S262L	7	Missense	Passenger	NA		12.32					before_last_exon	passenger		predicted passenger
c.1405C>T	p.P469S	14	Missense	Passenger	NA		0.006					before_last_exon	passenger		predicted passenger
c.896T>A	p.L299Q	8	Missense	Passenger	NA	Serinc	28					before_last_exon	passenger		predicted passenger
c.1816_1824dupGCCGGCCTC	p.A606_L608dupAGL	4	InFrameInsertion	Passenger	NA		5.802					last_portion	passenger		predicted passenger
c.3278G>A	p.R1093H	27	Missense	Passenger	NA	Ion_trans	23.4					before_last_exon	passenger		predicted passenger
c.2108G>A	p.R703H	18	Missense	Passenger	NA	Glyco_hydro_38C	16.85					before_last_exon	passenger		predicted passenger
c.932A>G	p.N311S	5	Missense	Passenger	NA		18.79					before_last_exon	passenger		predicted passenger
c.1142T>A	p.L381*	2	Nonsense	Passenger	NA		39					before_last_portion	passenger		predicted passenger
c.112G>C	p.G38R	2	Missense	Passenger	NA		0.048					before_last_portion	passenger		predicted passenger
c.817G>A	p.G273R	2	Missense	Passenger	NA	ART	27.4					before_last_exon	passenger		predicted passenger
c.401C>A	p.P134H	6	Missense	Passenger	NA		16.28					before_last_exon	passenger		predicted passenger
c.74T>A	p.L25Q	1	Missense	Passenger	NA	Bcr-Abl_Oligo	23.8		Act			before_last_exon	passenger		predicted passenger
c.1169G>A	p.R390H	2	Missense	Passenger	NA		29.5					last_portion	passenger		predicted passenger
c.1174C>T	p.R392C	10	Missense	Passenger	NA		27.6					before_last_exon	passenger		predicted passenger
c.10186C>T	p.R3396*	71	Nonsense	Passenger	NA		50					before_last_exon	passenger		predicted passenger
c.1289C>T	p.A430V	8	Missense	Passenger	NA	Asp	6.389					before_last_exon	passenger		predicted passenger
c.965G>A	p.G322D	8	Missense	Passenger	NA	Peptidase_M1	27.3					before_last_exon	passenger		predicted passenger
c.2533dupC	p.R845Pfs*3	10	Frameshift	Driver	Yes		24.4	tumor_driver	LoF			before_last_exon	TIER 1	predicted	predicted driver: tier 1
c.2863G>A	p.A955T	24	Missense	Driver	Yes	Pkinase_Tyr	32	other_tumor_driver	Act			before_last_exon	TIER 1	predicted	predicted driver: tier 1
c.872C>T	p.T291I	4	Missense	Passenger	NA		12.09					before_last_exon	passenger		predicted passenger
c.2130G>T	p.E710D	1	Missense	Passenger	NA		9.935					last_portion	passenger		predicted passenger
c.1066G>A	p.E356K	2	Missense	Driver	Yes		27.5	other_tumor_driver	ambiguous	in_cluster		before_last_portion	TIER 2	predicted	predicted driver: tier 2
c.76G>A	p.G26S	1	Missense	Passenger	NA	Chorein_N	28.9					before_last_exon	passenger		predicted passenger
c.1931G>C	p.G644A	19	Missense	Passenger	NA		28.5					before_last_exon	passenger		predicted passenger
c.494A>G	p.D165G	3	Missense	Passenger	NA	RRM_1	21.7	other_tumor_driver	ambiguous			before_last_exon	passenger		predicted passenger
c.1870C>T	p.H624Y	13	Missense	Passenger	NA		9.459					before_last_exon	passenger		predicted passenger
c.8293G>A	p.G2765S	57	Missense	Driver	Yes	PI3_PI4_kinase	33	tumor_driver	LoF		in_delicate_domain	before_last_exon	TIER 1	predicted	predicted driver: tier 1
c.298C>T	p.R100W	5	Missense	Passenger	NA		35					before_last_exon	passenger		predicted passenger
c.2186T>C	p.F729S	1	Missense	Passenger	NA		26.8		LoF			before_last_exon	passenger		predicted passenger
c.911T>C	p.M304T	10	Missense	Passenger	NA	TMEM131_like	22.7					before_last_exon	passenger		predicted passenger
c.918C>A	p.C306*	6	Nonsense	Passenger	NA		39					before_last_exon	passenger		predicted passenger
c.910T>A	p.Y304N	8	Missense	Passenger	NA	Reprolysin_5	22.6					before_last_exon	passenger		predicted passenger
c.448G>A	p.D150N	4	Missense	Passenger	NA	Myotub-related	23					before_last_exon	passenger		predicted passenger
c.2718G>T	p.K906N	19	Missense	Passenger	NA	Ig_2	26.1					before_last_exon	passenger		predicted passenger
c.4466C>T	p.P1489L	16	Missense	Passenger	NA	CHAT	28.3					before_last_exon	passenger		predicted passenger
c.1736T>G	p.L579R	11	Missense	Passenger	NA		26					before_last_portion	passenger		predicted passenger
c.1339C>T	p.R447W	11	Missense	Passenger	NA		28.5					before_last_exon	passenger		predicted passenger
c.3361G>A	p.G1121R	29	Missense	Passenger	NA		26.5					before_last_exon	passenger		predicted passenger
c.278A>G	p.E93G	7	Missense	Passenger	NA	A_deaminase	31					before_last_exon	passenger		predicted passenger
c.2123G>A	p.R708Q	7	Missense	Passenger	NA		24.9					last_portion	passenger		predicted passenger
c.1229G>A	p.R410Q	8	Missense	Passenger	NA	Peptidase_M2	13.84					before_last_exon	passenger		predicted passenger
c.3173G>A	p.G1058D	25	Missense	Driver	Yes		25.3	tumor_driver	Act			last_portion	TIER 1	predicted	predicted driver: tier 1
c.683C>T	p.A228V	1	Missense	Passenger	NA		23.3					before_last_portion	passenger		predicted passenger
c.1570G>A	p.V524I	2	Missense	Passenger	NA	LRR_8	2.912					before_last_portion	passenger		predicted passenger
c.511A>G	p.I171V	5	Missense	Passenger	NA		13.94	other_tumor_driver	LoF			before_last_exon	passenger		predicted passenger
c.229T>C	p.Y77H	1	Missense	Passenger	NA		26.5					before_last_exon	passenger		predicted passenger
c.205G>T	p.A69S	3	Missense	Passenger	NA	TIG	12.85					before_last_exon	passenger		predicted passenger
c.844G>A	p.G282S	10	Missense	Passenger	NA		34					before_last_exon	passenger		predicted passenger
c.5768C>T	p.A1923V	37	Missense	Passenger	NA		29.8					last_portion	passenger		predicted passenger
c.742A>T	p.K248*	7	Nonsense	Passenger	NA	Na_K-ATPase	44					before_last_portion	passenger		predicted passenger
c.185T>A	p.L62Q	1	Missense	Passenger	NA		26.9					before_last_exon	passenger		predicted passenger
c.2209G>C	p.V737L	19	Missense	Passenger	NA		22.7					before_last_exon	passenger		predicted passenger
c.490G>T	p.A164S	1	Missense	Passenger	NA		15.41					before_last_exon	passenger		predicted passenger
c.359_364delGTACTC	.	6	SpliceAcceptorDeletion	Passenger	NA		17.09					-	passenger		predicted passenger
c.1148G>A	p.R383H	7	Missense	Passenger	NA	UCH	15.06					before_last_exon	passenger		predicted passenger
c.2173C>T	p.R725C	16	Missense	Passenger	NA		35					before_last_exon	passenger		predicted passenger
c.3208C>G	p.L1070V	27	Missense	Passenger	NA		23.5					before_last_exon	passenger		predicted passenger
c.836G>A	p.G279D	5	Missense	Passenger	NA		6.493					before_last_exon	passenger		predicted passenger
c.485T>G	p.L162R	5	Missense	Driver	Yes	zf-H2C2_2	29.3	other_tumor_driver	ambiguous			before_last_exon	TIER 2	predicted	predicted driver: tier 2
c.139G>A	p.A47T	2	Missense	Passenger	NA	COX14	31					before_last_portion	passenger		predicted passenger
c.446A>T	p.Y149F	1	Missense	Passenger	NA	7tm_4	21.9					before_last_portion	passenger		predicted passenger
c.1219G>C	p.V407L	10	Missense	Passenger	NA	cNMP_binding	25.4					before_last_exon	passenger		predicted passenger
c.1483G>A	p.A495T	13	Missense	Passenger	NA		24.1	other_tumor_driver	LoF			before_last_portion	passenger		predicted passenger
c.802C>T	p.R268W	4	Missense	Passenger	NA	GST_C_3	26.4					before_last_exon	passenger		predicted passenger
c.1421A>T	p.N474I	8	Missense	Passenger	NA	AA_permease	11.65					before_last_exon	passenger		predicted passenger
c.598C>G	p.H200D	1	Missense	Passenger	NA		16.38		LoF			before_last_portion	passenger		predicted passenger
c.3250G>A	p.V1084I	18	Missense	Passenger	NA	Na_trans_assoc	5.656					before_last_exon	passenger		predicted passenger
c.796C>T	p.R266W	3	Missense	Passenger	NA		34					before_last_exon	passenger		predicted passenger
c.100C>T	p.R34W	1	Missense	Passenger	NA	EF-hand_7	23.7					before_last_portion	passenger		predicted passenger
c.842A>G	p.D281G	8	Missense	Driver	Yes	P53	31	tumor_driver	LoF	in_cluster		before_last_exon	TIER 1	known	known in: CANCER-PR
c.2161G>A	p.E721K	16	Missense	Passenger	NA		33					before_last_exon	passenger		predicted passenger
c.430C>T	p.R144*	2	Nonsense	Passenger	NA	RnaseA	23.3					before_last_portion	passenger		predicted passenger
c.193G>A	p.E65K	4	Missense	Passenger	NA		28.8					before_last_exon	passenger		predicted passenger
c.2359G>T	p.V787L	8	Missense	Passenger	NA		22	other_tumor_driver	LoF			before_last_exon	passenger		predicted passenger
c.1126G>A	p.A376T	14	Missense	Passenger	NA	FGGY_C	34					before_last_exon	passenger		predicted passenger

c.1417A>G	p.N473D	12	Missense	Passenger	NA	Laminin_EGF	10.95					before_last_exon	passenger		predicted passenger
c.2035G>C	p.E679Q	1	Missense	Passenger	NA		15.34					before_last_exon	passenger		predicted passenger
c.548G>A	p.R183Q	4	Missense	Driver	Yes	G-alpha	35	other_tumor_driver	Act			before_last_exon	TIER 1	predicted	predicted driver: tier 1
c.2574C>A	p.N858K	2	Missense	Passenger	NA	Protocadherin	22.1					before_last_exon	passenger		predicted passenger
c.598C>T	p.R200C	7	Missense	Passenger	NA		24.1					before_last_exon	passenger		predicted passenger
c.893C>T	p.T298M	2	Missense	Passenger	NA		18.16					last_portion	passenger		predicted passenger
c.7541_7542delCT	p.P2514Rfs*4	34	Frameshift	Driver	Yes	DUF3454	35	tumor_driver	ambiguous	in_cluster		last_portion	TIER 2	predicted	predicted driver: tier 2
c.9370G>A	p.E3124K	13	Missense	Passenger	NA		16.29	other_tumor_driver	LoF			before_last_exon	passenger		predicted passenger
c.941A>C	p.E314A	7	Missense	Driver	Yes		29.2	other_tumor_driver	Act			before_last_exon	TIER 2	predicted	predicted driver: tier 2
c.698G>C	p.G233A	8	Missense	Passenger	NA	Lung_7-TM_R	5.247					before_last_exon	passenger		predicted passenger
c.2614G>A	p.A872T	16	Missense	Driver	Yes		25.4	other_tumor_driver	LoF			before_last_exon	TIER 2	predicted	predicted driver: tier 2
c.2098A>G	p.K700E	15	Missense	Driver	Yes		28	tumor_driver	Act	in_cluster		before_last_exon	TIER 1	known	known in: AML
c.1091C>G	p.P364R	10	Missense	Passenger	NA		23.8					before_last_exon	passenger		predicted passenger
c.1237G>A	p.D413N	11	Missense	Passenger	NA	SIM_C	25.5					before_last_exon	passenger		predicted passenger
c.1639delC	p.Q547Nfs*21	9	Frameshift	Driver	Yes		35	tumor_driver	ambiguous			before_last_portion	TIER 2	predicted	predicted driver: tier 2
c.232T>C	p.Y78H	4	Missense	Passenger	NA		23.8					before_last_exon	passenger		predicted passenger
c.880G>C	p.V294L	5	Missense	Passenger	NA	Neur_chan_memb	23.9					before_last_exon	passenger		predicted passenger
c.1223G>A	p.R408Q	4	Missense	Passenger	NA	zf-H2C2_2	2.061					before_last_portion	passenger		predicted passenger
c.14798C>G	p.A4933G	101	Missense	Passenger	NA	E3_UbLigase_R4	34					before_last_exon	passenger		predicted passenger
c.2540C>T	p.T847M	21	Missense	Passenger	NA		24.4					before_last_exon	passenger		predicted passenger
c.794T>C	p.L265P	5	Missense	Driver	Yes	TIR	32	tumor_driver	Act			before_last_portion	TIER 1	known	known in: LY;WM
c.308T>G	p.V103G	4	Missense	Passenger	NA	bcl-2I13	23		LoF			before_last_exon	passenger		predicted passenger
c.724C>T	p.L242F	9	Missense	Passenger	NA	Dak1	26.4					before_last_exon	passenger		predicted passenger
c.1363C>T	p.R455*	13	Nonsense	Passenger	NA		38	other_tumor_driver	Act			before_last_exon	passenger		predicted passenger
c.1951C>T	p.P651S	7	Missense	Passenger	NA		24.4					before_last_portion	passenger		predicted passenger
c.95A>T	p.E32V	2	Missense	Passenger	NA		23.3					before_last_exon	passenger		predicted passenger
c.2251C>T	p.R751W	22	Missense	Passenger	NA	IQ	34					before_last_exon	passenger		predicted passenger
c.118C>T	p.R40W	1	Missense	Passenger	NA		27.8					before_last_portion	passenger		predicted passenger
c.811G>T	p.G271C	2	Missense	Passenger	NA	zf-H2C2_2	25.9					before_last_portion	passenger		predicted passenger
c.68A>G	p.H23R	2	Missense	Passenger	NA		0.001					before_last_exon	passenger		predicted passenger
c.645G>T	p.M215I	2	Missense	Passenger	NA	Homeobox	26				in_delicate_domain	before_last_portion	passenger		predicted passenger
c.1108G>A	p.A370T	11	Missense	Passenger	NA	IMPDH	24.1					before_last_exon	passenger		predicted passenger
c.1475C>G	p.T492S	5	Missense	Passenger	NA	VWA	8.315					before_last_exon	passenger		predicted passenger
c.1471G>C	p.D491H	5	Missense	Passenger	NA	VWA	22.8					before_last_exon	passenger		predicted passenger
c.24_34delGCCCCAGGACT	p.P9Sfs*28	1	Frameshift	Passenger	NA		22.8					before_last_portion	passenger		predicted passenger
c.660T>G	p.S220R	4	Missense	Passenger	NA	AMP-binding	25.8					before_last_exon	passenger		predicted passenger
c.190C>T	p.L64F	3	Missense	Passenger	NA	Ras	28.3					before_last_exon	passenger		predicted passenger
c.1685_1701delAAGCTAAACAAGAAGTG	p.E562Afs*10	15	Frameshift	Driver	Yes		35	tumor_driver	LoF			before_last_exon	TIER 1	predicted	predicted driver: tier 1
c.853A>G	p.T285A	5	Missense	Passenger	NA		25.9		Act			last_portion	passenger		predicted passenger
c.655G>C	p.A219P	5	Missense	Passenger	NA	Amidinotransf	28.9					before_last_exon	passenger		predicted passenger
c.2462T>C	p.V821A	21	Missense	Passenger	NA		5.912					before_last_exon	passenger		predicted passenger
c.620G>A	p.R207H	5	Missense	Driver	Yes		28.1	other_tumor_driver	Act			before_last_exon	TIER 2	predicted	predicted driver: tier 2
c.547C>T	p.H183Y	1	Missense	Passenger	NA	7tm_4	25.8					before_last_portion	passenger		predicted passenger
c.3134C>T	p.A1045V	18	Missense	Passenger	NA		15.21					before_last_exon	passenger		predicted passenger
c.1348C>A	p.L450I	14	Missense	Passenger	NA		24.6		Act			before_last_portion	passenger		predicted passenger
c.639C>A	p.S213R	7	Missense	Passenger	NA	Pkinase	24.1					before_last_exon	passenger		predicted passenger
c.5292A>C	p.R1764S	33	Missense	Passenger	NA		18.32		Act			before_last_exon	passenger		predicted passenger
c.2620C>T	p.P874S	34	Missense	Passenger	NA		24.5	other_tumor_driver	LoF			last_portion	passenger		predicted passenger
c.397G>A	p.V133M	3	Missense	Passenger	NA		23.4					before_last_exon	passenger		predicted passenger
c.7G>C	p.E3Q	2	Missense	Passenger	NA		24.9					before_last_exon	passenger		predicted passenger
c.529C>A	p.P177T	5	Missense	Passenger	NA		24.5		LoF			before_last_exon	passenger		predicted passenger
c.1132A>G	p.M378V	9	Missense	Passenger	NA		17.37		Act			before_last_exon	passenger		predicted passenger
c.5410C>T	p.R1804W	32	Missense	Passenger	NA	fn3	26.5					before_last_exon	passenger		predicted passenger
c.4968_4975delGCTGCCCA	p.Q1656Hfs*103	24	Frameshift	Passenger	NA		34					last_portion	passenger		predicted passenger
c.2563C>T	p.R855*	16	Nonsense	Passenger	NA		40		LoF			before_last_exon	passenger		predicted passenger
c.993_1006delGGAAATGCAGCCAT	p.M331Ifs*4	8	Frameshift	Passenger	NA	E1-E2_ATPase	35					before_last_exon	passenger		predicted passenger
c.2193G>T	p.R731S	14	Missense	Passenger	NA	Biotin_carb_C	25.6					before_last_exon	passenger		predicted passenger
c.5842G>T	p.V1948L	23	Missense	Passenger	NA	Cadherin	12.9					before_last_exon	passenger		predicted passenger
c.383G>A	p.G128D	1	Missense	Passenger	NA		22.8					last_portion	passenger		predicted passenger
c.2462G>A	p.R821Q	37	Missense	Passenger	NA	Collagen	24		Act			before_last_exon	passenger		predicted passenger
c.2110A>T	p.I704F	15	Missense	Driver	Yes		32	tumor_driver	Act			before_last_exon	TIER 1	predicted	predicted driver: tier 1
c.130C>A	p.P44T	3	Missense	Passenger	NA	Vinculin	19.33					before_last_exon	passenger		predicted passenger
c.114C>G	p.S38R	6	Missense	Driver	Yes	POT1	27.7	tumor_driver	ambiguous			before_last_exon	TIER 1	predicted	predicted driver: tier 1
c.7225C>T	p.Q2409*	34	Nonsense	Driver	No		41	tumor_driver	ambiguous			before_last_portion	TIER 2	predicted	predicted driver: tier 2
c.2084C>A	p.P695H	15	Missense	Passenger	NA		9.386		Act			before_last_exon	passenger		predicted passenger
c.451C>T	p.R151*	3	Nonsense	Passenger	NA	zf-H2C2_2	24.6					before_last_portion	passenger		predicted passenger
c.47_58delTGGAGTACGAGC	p.L16_E19delLEYE	1	InFrameDeletion	Passenger	NA		22.7	other_tumor_driver	LoF			before_last_exon	passenger		predicted passenger
c.1061G>A	p.R354H	10	Missense	Passenger	NA		0.079					before_last_exon	passenger		predicted passenger
c.790A>G	p.I264V	5	Missense	Passenger	NA		0.023					before_last_exon	passenger		predicted passenger
c.1371A>G	p.I457M	7	Missense	Passenger	NA		13.46					before_last_exon	passenger		predicted passenger
c.3733G>A	p.A1245T	25	Missense	Passenger	NA		25		Act			before_last_exon	passenger		predicted passenger
c.318C>G	p.S106R	4	Missense	Driver	Yes	P53	12.67	tumor_driver	LoF			before_last_exon	passenger	known	known in: CANCER-PR
c.1429C>T	p.R477C	9	Missense	Passenger	NA	Tektin	35					last_portion	passenger		predicted passenger
c.7541_7542delCT	p.P2514Rfs*4	34	Frameshift	Driver	Yes	DUF3454	35	tumor_driver	ambiguous	in_cluster		last_portion	TIER 2	predicted	predicted driver: tier 2
c.413G>C	p.R138P	4	Missense	Passenger	NA	RRM_1	25.9					before_last_exon	passenger		predicted passenger
c.2223G>C	p.K741N	15	Missense	Driver	Yes		29.4	tumor_driver	Act	in_cluster		before_last_exon	TIER 1	predicted	predicted driver: tier 1
c.2698C>T	p.R900W	12	Missense	Passenger	NA		23.6					before_last_exon	passenger		predicted passenger
c.2014C>T	p.R672W	15	Missense	Passenger	NA	SH3_9	33					before_last_portion	passenger		predicted passenger
c.1196C>G	p.A399G	1	Missense	Passenger	NA		0.163					before_last_portion	passenger		predicted passenger
c.388G>A	p.V130M	3	Missense	Passenger	NA	TNF	24.2					before_last_portion	passenger		predicted passenger
c.956C>T	p.A319V	5	Missense	Driver	Yes	GATA	34	other_tumor_driver	LoF			before_last_exon	TIER 1	predicted	predicted driver: tier 1
c.758T>G	p.L253W	3	Missense	Passenger	NA	DIE2_ALG10	19.77					before_last_portion	passenger		predicted passenger
c.1516T>C	p.S506P	3	Missense	Passenger	NA		21.3					last_portion	passenger		predicted passenger
c.716G>A	p.S239N	7	Missense	Passenger	NA	SEA	21.9					before_last_exon	passenger		predicted passenger
c.4084G>A	p.E1362K	29	Missense	Passenger	NA		23.3					before_last_exon	passenger		predicted passenger
c.89C>T	p.P30L	2	Missense	Driver	Yes	Hist_deacetyl	33	other_tumor_driver	LoF			before_last_exon	TIER 1	predicted	predicted driver: tier 1
c.259G>T	p.D87Y	1	Missense	Passenger	NA		13.78					before_last_exon	passenger		predicted passenger
c.204G>T	p.W68C	3	Missense	Passenger	NA	MAPEG	31					before_last_exon	passenger		predicted passenger
c.3442G>A	p.V1148M	22	Missense	Passenger	NA		29.5					before_last_exon	passenger		predicted passenger
c.652C>T	p.R218*	8	Nonsense	Passenger	NA		38					before_last_exon	passenger		predicted passenger
c.524G>A	p.R175K	5	Missense	Passenger	NA	FYTT	20.1					before_last_exon	passenger		predicted passenger
c.871G>A	p.D291N	10	Missense	Passenger	NA		22.8					before_last_exon	passenger		predicted passenger
c.1526C>G	p.S509W	14	Missense	Passenger	NA		7.898					before_last_portion	passenger		predicted passenger
c.837_845delGGCGGGTAT	p.A280_I282delAGI	9	InFrameDeletion	Passenger	NA	Myosin_head	20.2					before_last_exon	passenger		predicted passenger
c.767G>A	p.S256N	6	Missense	Passenger	NA	TruD	19.14					before_last_exon	passenger		predicted passenger
c.249G>T	p.M83I	3	Missense	Passenger	NA		24.3					before_last_exon	passenger		predicted passenger

c.1078G>A	p.A360T	2	Missense	Passenger	NA	Ion_trans	28.4				before_last_exon	passenger		predicted passenger
c.717G>T	p.K239N	7	Missense	Passenger	NA		26.7				before_last_exon	passenger		predicted passenger
c.2278G>A	p.V760M	25	Missense	Passenger	NA	HAD	24.4		LoF		before_last_exon	passenger		predicted passenger
c.1478C>G	p.A493G	2	Missense	Passenger	NA		25.5				before_last_portion	passenger		predicted passenger
c.130G>C	p.G44R	2	Missense	Driver	Yes		26.4	tumor_driver	Act		before_last_exon	TIER 1	predicted	predicted driver: tier 1
c.206C>A	p.A69D	5	Missense	Passenger	NA	AAA_18	10.5				before_last_exon	passenger		predicted passenger
c.1259G>A	p.R420H	10	Missense	Passenger	NA	Peptidase_S8	33				before_last_exon	passenger		predicted passenger
c.3519A>C	p.E1173D	26	Missense	Passenger	NA		4.725				last_portion	passenger		predicted passenger
c.1663_1666delAGAA	p.R555Hfs*12	9	Frameshift	Driver	Yes	zf-C3HC4_3	35	tumor_driver	ambiguous		before_last_portion	TIER 2	predicted	predicted driver: tier 2
c.107A>C	p.E36A	3	Missense	Passenger	NA	Porin_3	19.25				before_last_exon	passenger		predicted passenger
c.1575T>G	p.N525K	4	Missense	Passenger	NA		12.26				before_last_portion	passenger		predicted passenger
c.797G>A	p.R266Q	4	Missense	Passenger	NA	RRM_1	32				before_last_exon	passenger		predicted passenger
c.652G>A	p.V218I	7	Missense	Passenger	NA	Actin	10.14				before_last_exon	passenger		predicted passenger
c.372G>T	p.L124F	1	Missense	Passenger	NA	UDPGT	0.109	other_tumor_driver	ambiguous		before_last_exon	passenger		predicted passenger
c.560A>G	p.N187S	5	Missense	Passenger	NA	RRM_1	17.91				before_last_exon	passenger		predicted passenger
c.95C>T	p.A32V	2	Missense	Passenger	NA		28.6				before_last_exon	passenger		predicted passenger
c.35C>T	p.P12L	2	Missense	Passenger	NA		23.4				before_last_exon	passenger		predicted passenger
c.1718A>T	p.D573V	13	Missense	Passenger	NA		23.9				before_last_exon	passenger		predicted passenger
c.2338A>T	p.I780F	15	Missense	Driver	Yes		31	other_tumor_driver	LoF		before_last_exon	TIER 1	predicted	predicted driver: tier 1
c.86C>A	p.S29*	1	Nonsense	Passenger	NA	MIT	32				before_last_exon	passenger		predicted passenger
c.524dupA	p.D175Efs*7	6	Frameshift	Driver	Yes		34	other_tumor_driver	ambiguous		before_last_exon	TIER 2	predicted	predicted driver: tier 2
c.3157C>T	p.L1053F	24	Missense	Passenger	NA		10.76				before_last_exon	passenger		predicted passenger
c.5512G>A	p.A1838T	52	Missense	Passenger	NA		19.48				last_portion	passenger		predicted passenger
c.483G>T	p.E161D	3	Missense	Passenger	NA	MAGE	23				before_last_portion	passenger		predicted passenger
c.1186C>T	p.P396S	7	Missense	Passenger	NA	Peptidase_C98	25				before_last_exon	passenger		predicted passenger
c.759_762delTTAC	p.Y254Sfs*13	1	Frameshift	Driver	Yes			tumor_driver	ambiguous		before_last_exon	TIER 2	predicted	predicted driver: tier 2
c.74T>G	p.L25R	2	Missense	Passenger	NA	Ribosomal_S4	24.5				before_last_exon	passenger		predicted passenger
c.1366C>T	p.R456C	14	Missense	Passenger	NA		34				before_last_exon	passenger		predicted passenger
c.3203C>A	p.A1068E	26	Missense	Passenger	NA		25.7				before_last_exon	passenger		predicted passenger
c.470C>A	p.T157N	6	Missense	Passenger	NA		21.5				before_last_exon	passenger		predicted passenger
c.553G>A	p.A185T	1	Missense	Passenger	NA		12.27				before_last_exon	passenger		predicted passenger
c.1997A>G	p.K666R	14	Missense	Driver	Yes		23.5	tumor_driver	Act	in_cluster	before_last_exon	TIER 1	known	known in: AML
c.1180G>A	p.E394K	9	Missense	Passenger	NA	Trypsin	17.64				before_last_exon	passenger		predicted passenger
c.4444A>G	p.S1482G	32	Missense	Passenger	NA	Myosin_tail_1	15.36				before_last_exon	passenger		predicted passenger
c.1256G>A	p.R419Q	10	Missense	Passenger	NA		25.4				before_last_exon	passenger		predicted passenger
c.695T>C	p.M232T	4	Missense	Driver	Yes	TIR	24.6	tumor_driver	Act	in_cluster	before_last_exon	TIER 1	predicted	predicted driver: tier 1
c.442C>T	p.P148S	4	Missense	Passenger	NA	Neur_chan_LBD	26.9				before_last_exon	passenger		predicted passenger
c.3197A>C	p.K1066T	26	Missense	Passenger	NA		2.914				before_last_exon	passenger		predicted passenger
c.475C>T	p.R159C	1	Missense	Passenger	NA		23.4				before_last_portion	passenger		predicted passenger
c.4940G>T	p.S1647I	5	Missense	Passenger	NA		23.6				before_last_exon	passenger		predicted passenger
c.808G>T	p.A270S	9	Missense	Passenger	NA		23.9				before_last_exon	passenger		predicted passenger
c.265G>T	p.A89S	3	Missense	Passenger	NA	Serpin	24				before_last_exon	passenger		predicted passenger
c.32A>G	p.H11R	2	Missense	Passenger	NA		0.001				before_last_exon	passenger		predicted passenger
c.4006C>T	p.R1336C	5	Missense	Passenger	NA	Cadherin	34				before_last_exon	passenger		predicted passenger
c.1382A>G	p.K461R	5	Missense	Passenger	NA		17.66				before_last_portion	passenger		predicted passenger
c.1364C>G	p.P455R	5	Missense	Passenger	NA	zf-H2C2_2	24.9	other_tumor_driver	Act		before_last_portion	passenger		predicted passenger
c.41A>G	p.E14G	2	Missense	Passenger	NA		11.49				before_last_exon	passenger		predicted passenger
c.28C>T	p.R10C	3	Missense	Passenger	NA		11.59				before_last_portion	passenger		predicted passenger
c.335G>A	p.S112N	1	Missense	Passenger	NA	GAF	12.63				before_last_exon	passenger		predicted passenger
c.286T>C	p.S96P	2	Missense	Passenger	NA	HLH	23.3				before_last_exon	passenger		predicted passenger
c.103G>A	p.E35K	1	Missense	Passenger	NA	Cyclin_N	31		Act		before_last_exon	passenger		predicted passenger
c.1406G>A	p.C469Y	1	Missense	Passenger	NA	SapB_2	23.4				before_last_portion	passenger		predicted passenger
c.446delA	p.E149Gfs*3	4	Frameshift	Passenger	NA	Tetraspannin	26.8				before_last_exon	passenger		predicted passenger
c.692A>G	p.Y231C	8	Missense	Passenger	NA	zf-DHHC	27.1				before_last_exon	passenger		predicted passenger
c.4994G>A	p.R1665K	28	Missense	Passenger	NA		28.8				last_portion	passenger		predicted passenger
c.1060G>A	p.G354S	9	Missense	Passenger	NA	Rhomboid	32				before_last_portion	passenger		predicted passenger
c.1003C>G	p.R335G	9	Missense	Passenger	NA	RasGEF	15.64				before_last_exon	passenger		predicted passenger
c.58G>A	p.E20K	2	Missense	Driver	Yes	Not3	34	other_tumor_driver	Act		before_last_exon	TIER 1	predicted	predicted driver: tier 1
c.691C>T	p.P231S	7	Missense	Passenger	NA		17.2				before_last_exon	passenger		predicted passenger
c.1532A>G	p.N511S	2	Missense	Passenger	NA	Ank	0.236				before_last_portion	passenger		predicted passenger
c.1703T>A	p.V568E	9	Missense	Driver	Yes	zf-C3HC4_3	34.0	tumor_driver	Act		before_last_portion	TIER 1	predicted	predicted driver: tier 1
c.434G>A	p.R145H	4	Missense	Passenger	NA	THRAP3_BCLAF1	23.8	other_tumor_driver	Act		before_last_exon	passenger		predicted passenger
c.7541_7542delCT	p.P2514Rfs*4	34	Frameshift	Driver	Yes	DUF3454	35	tumor_driver	ambiguous	in_cluster	last_portion	TIER 2	predicted	predicted driver: tier 2
c.2057A>G	p.D686G	1	Missense	Passenger	NA		15.86				before_last_exon	passenger		predicted passenger
c.953T>A	p.I318N	12	Missense	Driver	Yes	XAP5	33	other_tumor_driver	ambiguous		before_last_exon	TIER 2	predicted	predicted driver: tier 2
c.10271C>T	p.T3424M	17	Missense	Driver	Yes	Cadherin	25.1	other_tumor_driver	LoF		before_last_exon	TIER 2	predicted	predicted driver: tier 2
c.401G>C	p.G134A	4	Missense	Driver	Yes		21.7	other_tumor_driver		in_cluster	before_last_portion	TIER 2	predicted	predicted driver: tier 2
c.3320C>T	p.P1107L	26	Missense	Passenger	NA		10.48				before_last_exon	passenger		predicted passenger
c.296C>T	p.T99M	1	Missense	Passenger	NA	PAP_central	24.3				before_last_portion	passenger		predicted passenger
c.8867C>T	p.S2956F	47	Missense	Passenger	NA	AAA_8	33				before_last_exon	passenger		predicted passenger
c.2042_2044delGAA	p.R681delR	12	InFrameDeletion	Passenger	NA		5.986				before_last_portion	passenger		predicted passenger
c.419G>A	p.R140Q	4	Missense	Passenger	NA	GIDE	34				before_last_portion	passenger		predicted passenger
c.2047G>A	p.G683S	16	Missense	Passenger	NA	COG7	34				before_last_exon	passenger		predicted passenger
c.230G>A	p.R77H	2	Missense	Passenger	NA	zf-C3HC4_2	34				before_last_exon	passenger		predicted passenger
c.1660G>A	p.D554N	1	Missense	Passenger	NA		24.2				before_last_portion	passenger		predicted passenger
c.1351G>T	p.A451S	15	Missense	Passenger	NA		0.251				before_last_exon	passenger		predicted passenger
c.293C>T	p.A98V	2	Missense	Passenger	NA	Laminin_G_2	26.8				before_last_exon	passenger		predicted passenger
c.524G>A	p.C175Y	4	Missense	Passenger	NA	Trypsin	26				before_last_exon	passenger		predicted passenger
c.431G>A	p.R144Q	4	Missense	Passenger	NA	CP2	35				before_last_exon	passenger		predicted passenger
c.1481A>C	p.K494T	13	Missense	Passenger	NA	TH1	23				before_last_exon	passenger		predicted passenger
c.589G>A	p.E197K	3	Missense	Passenger	NA		26.6				before_last_exon	passenger		predicted passenger
c.796C>G	p.L266V	4	Missense	Passenger	NA		20.8				before_last_portion	passenger		predicted passenger
c.431T>C	p.F144S	3	Missense	Passenger	NA	Pep_M12B_propep	24.4		LoF		before_last_exon	passenger		predicted passenger
c.1184delG	p.G395Efs*5	14	Frameshift	Driver	Yes	KH_1	27.5	other_tumor_driver	LoF		before_last_exon	TIER 1	predicted	predicted driver: tier 1
c.1787A>G	p.H596R	18	Missense	Passenger	NA	Carn_acyltransf	10.12				before_last_exon	passenger		predicted passenger
c.1529C>T	p.T510M	7	Missense	Passenger	NA		19.71				before_last_portion	passenger		predicted passenger
c.1750C>T	p.P584S	15	Missense	Passenger	NA		26.2				before_last_exon	passenger		predicted passenger
c.247G>A	p.V83M	2	Missense	Passenger	NA		22.1				before_last_exon	passenger		predicted passenger
c.301C>T	p.H101Y	1	Missense	Passenger	NA	Laminin_N	28.8				before_last_exon	passenger		predicted passenger
c.508G>A	p.A170T	1	Missense	Passenger	NA	7tm_4	19.15				before_last_portion	passenger		predicted passenger
c.851T>A	p.V284E	5	Missense	Passenger	NA		34		Act		last_portion	passenger		predicted passenger
c.2766A>T	p.E922D	28	Missense	Passenger	NA		17.73		Act		before_last_exon	passenger		predicted passenger
c.200C>T	p.P67L	2	Missense	Passenger	NA	MIP	29				before_last_exon	passenger		predicted passenger
c.1369C>T	p.R457W	11	Missense	Passenger	NA		24.1				before_last_exon	passenger		predicted passenger
c.9194G>A	p.G3065D	58	Missense	Passenger	NA	Sushi	26.9				before_last_exon	passenger		predicted passenger
c.1949C>T	p.P650L	1	Missense	Passenger	NA	Cadherin	22.7		LoF		before_last_exon	passenger		predicted passenger

c.365A>G	p.K122R	2	Missense	Passenger	NA	Tau95	18.39			before_last_exon	passenger		predicted passenger
c.692C>T	p.T231M	1	Missense	Passenger	NA	Cadherin	1.637			before_last_exon	passenger		predicted passenger
c.170delT	p.L57Pfs*48	3	Frameshift	Passenger	NA		23.2			before_last_exon	passenger		predicted passenger
c.751G>A	p.E251K	8	Missense	Passenger	NA		33			before_last_exon	passenger		predicted passenger
c.4201C>T	p.Q1401*	18	Nonsense	Driver	Yes		39	tumor_driver	LoF	before_last_exon	TIER 1	predicted	predicted driver: tier 1
c.778A>T	p.S260C	5	Missense	Passenger	NA		5.413			before_last_portion	passenger		predicted passenger
c.1010G>A	p.R337H	5	Missense	Passenger	NA	Aminotran_5	23.5			before_last_exon	passenger		predicted passenger
c.759_762delTTAC	p.Y254Sfs*13	1	Frameshift	Driver	Yes			tumor_driver	ambiguous	before_last_exon	TIER 2	predicted	predicted driver: tier 2
c.164_166delAGG	p.E55delE	1	InFrameDeletion	Passenger	NA		10.04			before_last_exon	passenger		predicted passenger
c.326C>A	p.T109N	4	Missense	Passenger	NA	Transferrin	22.3		LoF	before_last_exon	passenger		predicted passenger
c.484G>A	p.A162T	3	Missense	Passenger	NA	Ank_2	25.1			before_last_exon	passenger		predicted passenger
c.142C>T	p.R48W	2	Missense	Passenger	NA		33			before_last_exon	passenger		predicted passenger
c.125T>A	p.I42N	3	Missense	Passenger	NA		11.4			before_last_exon	passenger		predicted passenger
c.949C>G	p.H317D	12	Missense	Driver	Yes	XAP5	32	other_tumor_driver	ambiguous	before_last_exon	TIER 2	predicted	predicted driver: tier 2
c.1766A>G	p.E589G	9	Missense	Passenger	NA		23.6	other_tumor_driver	Act	before_last_exon	passenger		predicted passenger
c.3593C>T	p.P1198L	50	Missense	Passenger	NA	Collagen	26.6		Act	before_last_exon	passenger		predicted passenger
c.9913G>T	p.E3305*	50	Nonsense	Passenger	NA		39			before_last_exon	passenger		predicted passenger
c.1397G>A	p.R466H	5	Missense	Passenger	NA		34			before_last_exon	passenger		predicted passenger
