# Supplementary material for: CLL cells cumulate genetic aberrations prior to the first therapy even in outwardly inactive disease phase
Source: Leukemia. 2018 Sep 12;33(2):518–58. doi: 10.1038/s41375-018-0255-1 (PMC6756121; doi:10.1038/s41375-018-0255-1)
Supplement: Supplementary file 4 — Supplementary Table S3 [file 41375_2018_255_MOESM4_ESM.pdf]

Table S3: Driver mutations detected by WES and validated using DTS in CLL patients; selection based on *in silico* Cancer Genome Interpreter analysis

Patient ID	Group of patients	Acquired chromosomal alteration at TP2	Gene	cDNA change	Protein change	CLL known-driver (Landau et al., 2015; Puente et al., 2015)	Validated by TDS	Allele frequency in TP1	Allele frequency in TP2	Evolution p-value	Ratio TP2/TP1	Status at TP2
5	Indolent	No	*SF3B1*	c.1986C>G	p.H662Q	Yes	Yes	32.68	48.89	4.99E-08	1.50	Static
9	Indolent	No	*ERCC6*	c.625G>T	p.D209Y	No	Yes	22.58	38.59	1.47E-30	1.71	Increased
31	Indolent	Yes	*CCND1*	c.125T>G	p.V42G	Yes	Yes	9.83	15.63	1.49E-20	1.59	Increased
41	Indolent	Yes	*IDH1*	c.890G>A	p.C297Y	No	Yes	49.35	49.39	0.97412	1.00	Static
46	Indolent	No	*NOTCH1*	c.7541_7542del	p.P2514fs	Yes	New	16.41	32.87	1.23E-14	2.00	Increased
6	Stable	No	*CDC73*	c.571G>C	p.A191P	No	Yes	5.75	37.28	5E-324	6.48	Increased
6	Stable	No	*CDC73*	c.932C>G	p.T311S	No	Yes	33.12	56.76	1.13E-54	1.71	Increased
6	Stable	No	*NFKBIE*	c.759_762del	p.T253fs	Yes	New	8.22	16.57	2.20E-16	2.02	Increased
6	Stable	No	*SF3B1*	c.1997A>C	p.K666T	Yes	Yes	38.68	20.41	4.68E-16	0.53	Static
6	Stable	No	*XPO1*	c.1711G>A	p.E571K	Yes	New	6.47	2.43	9.56E-12	0.38	Static
32	Stable	Yes	*DHX9*	c.1347T>G	p.S449R	No	Yes	0.78	15.27	9.28E-192	19.58	Increased
32	Stable	Yes	*RPS15*	c.430C>T	p.H137Y	Yes	Yes	14.71	11.61	0.00016261	0.79	Static
35	Stable	No	*NFKBIE*	c.759_762del	p.T253fs	Yes	New	0.00	14.80	2.2E-16	-	Acquired
35	Stable	No	*RPS15*	c.391C>T	p.P131S	Yes	Yes	42.90	47.47	0.000368865	1.11	Static
42	Stable	Yes	*ATM*	c.8293G>A	p.G2765S	Yes	Yes	47.02	50.51	0.000454039	1.07	Static
42	Stable	Yes	*EGFR*	c.2863G>A	p.A955T	No	Yes	48.79	51.19	0.01072518	1.05	Static
42	Stable	Yes	*EGR2*	c.1066G>A	p.E356K	Yes	Yes	48.00	47.94	0.9589161	1.00	Static
42	Stable	Yes	*MLL2*	c.2533dupC	p.R845Pfs*3	Yes	Yes	34.02	35.07	0.4531495	1.03	Static
44	Stable	No	*SF3B1*	c.2098A>G	p.K700E	Yes	New	3.26	6.84	1.08E-10	2.10	Increased
44	Stable	No	*XPO1*	c.3173G>A	p.G1058D	Yes	Yes	14.31	53.70	5E-324	3.75	Increased
45	Stable	No	*IKZF3*	c.485T>G	p.L162R	Yes	Yes	0.00	26.44	5E-324	-	Acquired
1	Active	Yes	*GNAQ*	c.548G>A	p.R183Q	No	Yes	0.00	44.42	5E-324	-	Acquired
1	Active	Yes	*NOTCH1*	c.7541_7542delCT	p.P2514Rfs*4	Yes	Yes	44.82	58.98	2.46E-07	1.32	Static
1	Active	Yes	*TP53*	c.842A>G	p.D281G	Yes	Yes	0.00	72.73	5E-324	-	Acquired
3	Active	No	*CARD11*	c.941A>C	p.E314A	Yes	Yes	51.18	51.88	0.3961178	1.01	Static
3	Active	No	*DICER1*	c.2614G>A	p.A872T	No	Yes	49.69	49.57	0.8542839	1.00	Static
3	Active	No	*SF3B1*	c.2098A>G	p.K700E	Yes	Yes	42.99	45.20	0.06001248	1.05	Static
4	Active	Yes	*BIRC3*	c.1639delC	p.Q547Nfs*21	Yes	Yes	2.52	31.67	2.84E-303	12.57	Increased
7	Active	No	*MYD88*	c.794T>C	p.L265P	Yes	Yes	0.35	42.98	5E-324	122.80	Increased
10	Active	No	*DDX3X*	c.1685_1701delAAGCTAAACAAGAAGTG	p.E562Afs*10	Yes	Yes	17.62	81.57	5E-324	4.63	Increased
11	Active	No	*CARD11*	c.620G>A	p.R207H	Yes	Yes	75.81	47.11	8.54E-129	0.62	Static
11	Active	No	*SF3B1*	c.2110A>T	p.I704F	Yes	Yes	23.09	48.29	1.15E-65	2.09	Increased
12	Active	No	*POT1*	c.114C>G	p.S38R	Yes	Yes	48.77	42.55	0.000681789	0.87	Static
12	Active	No	*TP53*	c.390delC	p.L130fs	Yes	New	3.26	10.71	0.001957	3.29	Increased
15	Active	No	*NOTCH1*	c.7541_7542delCT	p.P2514Rfs*4	Yes	Yes	62.87	0.42	2.26E-176	0.01	Decreased
15	Active	No	*SF3B1*	c.2223G>C	p.K741N	Yes	Yes	23.71	23.19	0.6678381	0.98	Static
15	Active	No	*TP53*	c.318C>G	p.S106R	Yes	Yes	14.41	24.85	9.87E-20	1.72	Increased
17	Active	No	*GATA3*	c.956C>T	p.A319V	No	Yes	48.16	85.76	5E-324	1.78	Increased
17	Active	No	*HDAC2*	c.89C>T	p.P30L	No	Yes	32.75	46.23	4.39E-48	1.41	Static
24	Active	No	*BIRC3*	c.1663_1666delAGAA	p.R555Hfs*12	Yes	Yes	3.87	15.83	6.44E-111	4.09	Increased
24	Active	No	*MED12*	c.130G>C	p.G44R	Yes	Yes	26.71	16.56	1.84E-16	0.62	Static
33	Active	Yes	*G3BP1*	c.524dupA	p.D175Efs*7	No	Yes	48.97	49.49	0.5816296	1.01	Static
33	Active	Yes	*PTCH1*	c.2338A>T	p.I780F	No	Yes	48.41	51.17	0.001203579	1.06	Static
33	Active	Yes	*SF3B1*	c.2323C>G	p.R775G	Yes	New	8.24	13.18	1.741E-11	1.60	Increased
34	Active	No	*NFKBIE*	c.759_762del	p.T253fs	Yes	Yes	6.37	10.80	2.40E-17	1.70	Increased
34	Active	No	*SF3B1*	c.1997A>G	p.K666R	Yes	Yes	45.62	49.03	0.02285284	1.07	Static
37	Active	Yes	*MYD88*	c.695T>C	p.M232T	Yes	Yes	46.27	48.04	0.01704658	1.04	Static
38	Active	Yes	*BIRC3*	c.1703T>A	p.V568E	Yes	Yes	8.61	10.81	2.81E-07	1.26	Static
38	Active	Yes	*CNOT3*	c.58G>A	p.E20K	Yes	Yes	29.16	26.45	0.000507196	0.91	Static
38	Active	Yes	*FAM50A*	c.953T>A	p.I318N	Yes	Yes	32.84	38.64	2.67E-11	1.18	Static
38	Active	Yes	*FAT1*	c.10271C>T	p.T3424M	No	Yes	50.20	50.67	0.7352296	1.01	Static
38	Active	Yes	*NOTCH1*	c.7541_7542delCT	p.P2514Rfs*4	Yes	Yes	58.10	56.44	0.1179186	0.97	Static
39	Active	No	*FUBP1*	c.1184delG	p.G395Efs*5	Yes	Yes	50.07	49.93	0.8746446	1.00	Static
39	Active	No	*RPS15*	c.401G>C	p.G134A	Yes	Yes	49.39	46.66	0.009833043	0.94	Static
43	Active	Yes	*ARID1A*	c.4201C>T	p.Q1401*	Yes	Yes	8.20	40.55	3,952525E-323	4.95	Increased
43	Active	Yes	*FAM50A*	c.949C>G	p.H317D	Yes	Yes	31.29	99.59	5E-324	3.18	Increased
43	Active	Yes	*NFKBIE*	c.759_762del	p.T253fs	Yes	Yes	50.53	45,00*	NA	0.89	Static

*Acquired: significant p-value and Allele Frequency TP1=0
*Increased: significant p-value and >1,5
*Decrased: significant p-value and <0,375
*Static: non significant p-value

Abbreviations:
TP - time-point
TDS - targeted deep sequencing
NA - not available
